# HDP—A Novel Heme Detoxification Protein from the Malaria Parasite

**DOI:** 10.1371/journal.ppat.1000053

**Published:** 2008-04-25

**Authors:** Dewal Jani, Rana Nagarkatti, Wandy Beatty, Ross Angel, Carla Slebodnick, John Andersen, Sanjai Kumar, Dharmendar Rathore

**Affiliations:** 1 Virginia Bioinformatics Institute, Virginia Tech, Blacksburg, Virginia, United States of America; 2 Washington University School of Medicine, St. Louis, Missouri, United States of America; 3 Department of Geosciences, Virginia Tech, Blacksburg, Virginia, United States of America; 4 Department of Chemistry, Virginia Tech, Blacksburg, Virginia, United States of America; 5 Laboratory of Malaria and Vector Research, National Institutes of Health, Rockville, Maryland, United States of America; 6 Food and Drug Administration, Bethesda, Maryland, United States of America; Albert Einstein College of Medicine, United States of America

## Abstract

When malaria parasites infect host red blood cells (RBC) and proteolyze hemoglobin, a unique, albeit poorly understood parasite-specific mechanism, detoxifies released heme into hemozoin (Hz). Here, we report the identification and characterization of a novel *Plasmodium* Heme Detoxification Protein (HDP) that is extremely potent in converting heme into Hz. HDP is functionally conserved across *Plasmodium* genus and its gene locus could not be disrupted. Once expressed, the parasite utilizes a circuitous “Outbound–Inbound” trafficking route by initially secreting HDP into the cytosol of infected RBC. A subsequent endocytosis of host cytosol (and hemoglobin) delivers HDP to the food vacuole (FV), the site of Hz formation. As Hz formation is critical for survival, involvement of HDP in this process suggests that it could be a malaria drug target.

## Author Summary

Each year, more than one million people, most of them children under the age of 5, succumb to malaria, a devastating disease caused by *Plasmodium* parasites. The parasite resides inside the red blood cells of the host, where, during its development, it proteolyzes vast amounts of host hemoglobin. This degradation also releases heme, which is extremely toxic to the parasite. To protect itself (from the toxic effects of heme), the parasite converts free heme into hemozoin. This parasite-specific mechanism is widely accepted as the weakest link in its lifecycle and is targeted by several of the currently available antimalarial drugs, which prevent hemozoin formation by binding to heme. However, due to an incomplete understanding of the parasite processes that lead to hemozoin formation, a drug that specifically targets the parasite factors responsible for hemozoin production has never been developed. Here we identify and characterize Heme Detoxification Protein, a unique *Plasmodium* protein, which we show as the potent producer of hemozoin. We demonstrate that this protein is highly conserved across the *Plasmodium* genus, is extremely efficient in producing hemozoin, and is delivered to the food vacuole, the site of hemozoin formation, via a unique trafficking route. We also demonstrate the critical nature of this protein and suggest that it could be targeted to develop novel antimalarial drugs.

## Introduction

Malaria is the most lethal parasitic disease and a dominant public health issue in more than 100 nations. While malaria infection begins with the invasion of hepatocytes by the *Plasmodium* sporozoites inoculated by an infected mosquito, the common clinical symptoms of malaria, which includes high fever, chills and anemia, are due to the subsequent infection and rapid multiplication of the parasite inside the RBC. To sustain its rapid pace of development, the parasite digests host hemoglobin, which represents 90% of the total protein present inside an RBC [1]; approximately 75% of which is degraded during the erythrocytic stages of development [2]. While hydrolysis of hemoglobin makes amino acids available for parasite development, this process also releases its lipophilic prosthetic group–heme, which is extremely toxic to the parasite. Therefore, along with a continuous degradation of hemoglobin, a concomitant detoxification of heme is necessary for an uninterrupted growth and proliferation of the parasite. Parasite effectively detoxifies heme, primarily by its conversion into an insoluble crystalline material called Hz. It is estimated that up to 75% of the free heme is processed into Hz [3],[4]. In its human host, heme detoxification is one of the homeostasis processes, performed by a combination of proteins like hemopexin and heme oxygenase [5], whose homologs have not been found in the parasite genome.

Disruption of Hz formation is the most widely used strategy for controlling malaria (Reviewed in [6]). For example, chloroquine primarily acts by binding to free heme with 1:2 stoichiometry, which prevents its detoxification into Hz [7],[8]. Similarly, one of the antimalarial activities of artemisinin involves its interaction with heme, leading to the formation of heme adducts that cannot be detoxified [9]. While the parasite-specific nature of this process has led to the development of several more drugs that interact with heme (i.e. the substrate), due to an incomplete knowledge of parasite factors involved, antimalarials that can target the process itself, have not been developed. The underlying mechanism, though poorly understood, is believed to be highly conserved as Hz formation occurs in all the species of *Plasmodium* during their intraerythrocytic development, irrespective of the host species they infect.

To date, parasite factors responsible for Hz formation are a subject of intense debate. In vitro, a protein [10] and a lipid-driven [11],[12] processes for Hz synthesis have been described by several research groups. Additionally, an autocatalytic process, where preformed Hz promotes the conversion of free heme into Hz, has also been proposed [13]. While it's been argued that the currently known parasite factors are not the major force behind the Hz production activities of *Plasmodium* [14]–[16], others believe that lipids could be the primary mediator of Hz formation in the parasite [17],[18]. Here we describe HDP, a parasite protein, which is a potent producer of Hz and demonstrate that it reaches its intracellular destination utilizing a novel trafficking route that has not been seen for any of the known malaria proteins.

## Results

### Cloning, recombinant expression and purification of *P. falciparum* HDP

*P. falciparum* HDP is a single copy, three-exons encoded [19], 205 amino acids long polypeptide (PlasmoDB # PF14_0446; GenBank Acc# NP_702335; Fig. S1,S2). In *P. falciparum* parasites, we found that the HDP gene was actively transcribed during the intraerythrocytic stages of the lifecycle (Fig. S1A). To study its role in the lifecycle of the parasite, the coding sequence of HDP was cloned and expressed in *E. coli.* On expression, the recombinant protein was localized in the inclusion bodies from which it was purified to homogeneity, by a two-step column chromatography (Fig. S1C,D).

### HDP binds heme and converts it into Hemozoin

**Figure 1:**
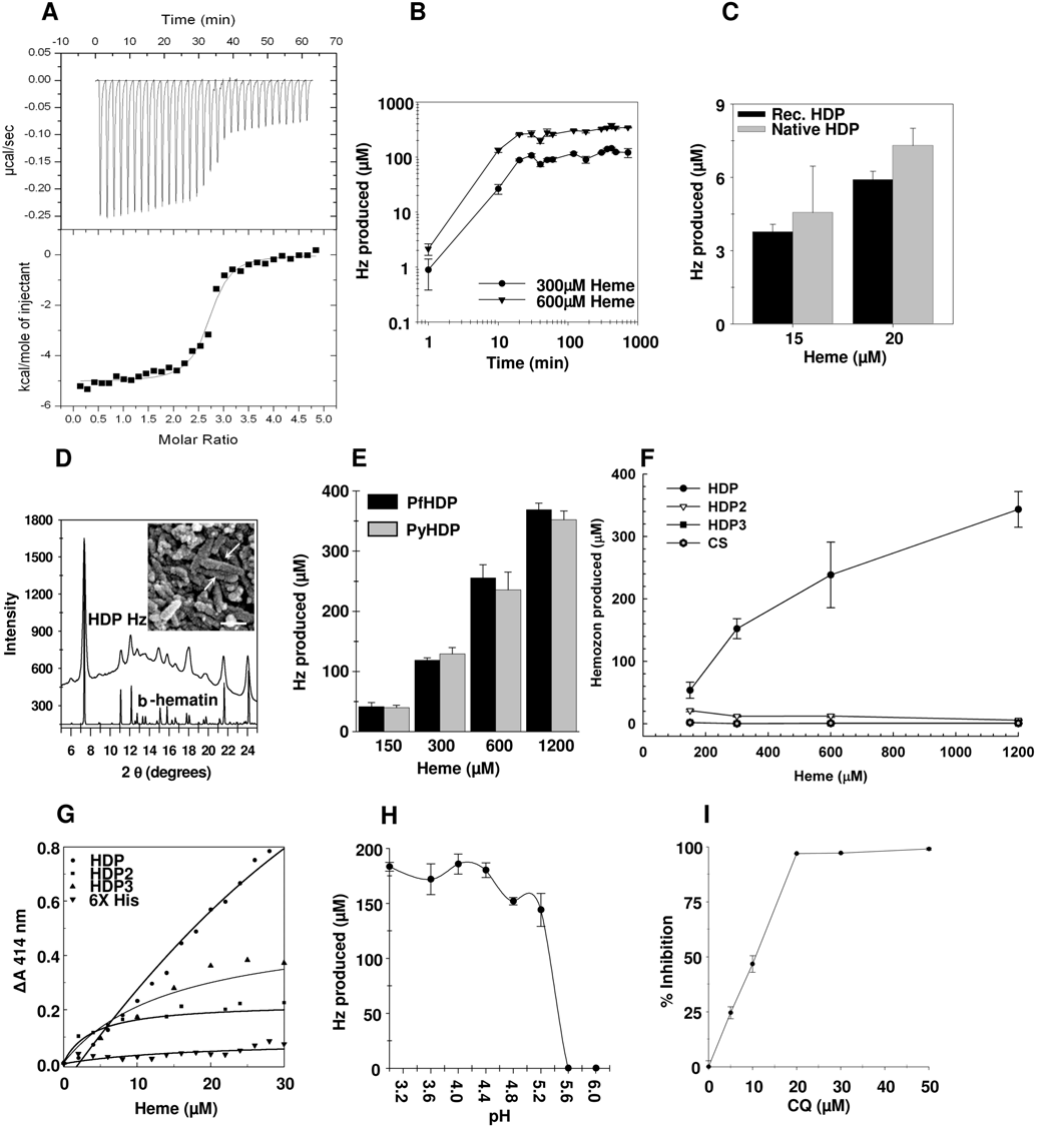
HDP rapidly detoxifies and sequesters heme as Hz. A, Heme (100 µM) solution was titrated into protein (5 µM) and the heat evolved was measured by Isothermal titration calorimetry. Binding isotherm was generated by integrating the data from the top panel. B, HDP-mediated Hz formation was concentration dependent and occurred rapidly with up to 75% of the total Hz being produced within the first 20 minutes. C, Native HDP (grey) can produce Hz at levels comparable to the recombinant protein (black). D, Powder X-ray diffraction pattern of HDP-produced Hz matches with that of β-hematin. Inset–Scanning electron microscopy image of HDP-produced Hz crystallites that are 0.1–0.2 µm in length (arrows) with a width of approx.0.05 µm (Bar, 0.1 µm) E, Hz formation activity of 0.5 µM *P. yoelii* HDP (grey bars) is indistinguishable from its *P. falciparum* ortholog, utilized at identical concentration (black bars). F, Full length HDP (closed circle) is necessary for Hz formation as HDP2 (open circle) and HDP3 (closed triangle) alone could not produce Hz. 0.5 µM of each of the three proteins was utilized for analysis. G, Heme in 2 µM increments was added to a 10 µM solution of either HDP, HDP2, HDP3 or polyhistidine peptide in 100 mM Sodium acetate buffer pH 5.2. Change in absorbance at 414 nm was plotted against the concentration of heme. The curve depicts the non-linear fit of the data. H, HDP-mediated Hz production is restricted to a pH range found inside the FV. I, CQ inhibits HDP-mediated Hz production. Values are mean±s. d

Purified HDP was utilized for investigating HDP-heme interactions. First, we measured the affinity of HDP towards heme by isothermal titration calorimetry (Fig. 1A). The analysis revealed that HDP binds heme with high affinity (*K*_d_ = 80 nM), and possesses 2.7 heme binding sites. Having established its ability to interact with heme, we evaluated HDP's potential to convert heme into Hz. In a Hz-production assay [10], where HDP was present in enzyme-like concentrations (0.5 µM) and heme at several hundred fold molar excess (300 and 600 µM), the protein rapidly converted up to 50% of heme into hemozoin, in a concentration dependent manner (Fig. 1B). HDP acted rapidly, generating most of the Hz within the first 20 minutes. The reaction achieved completion, typically within the first hour, showing little change in Hz levels over longer incubation periods (Fig. 1B). At the highest concentration of heme (600 µM) tested, HDP converted 261 µM of heme into Hz, leading to a conversion rate of 783 µM/hr/0.5 µM of protein. This translates into 1566 molecules of heme sequestered into Hz per hour/molecule of HDP (Fig. 1B). To explore if the activity of the recombinant HDP represents the function of the wild-type protein, we immunoprecipitated native HDP from the extracts of *P. falciparum*–infected RBCs. Protein-specific antibodies were raised using the recombinant HDP as an immunogen. The native protein was purified by immunoprecipitation from the infected RBC extracts (Fig. S7B) and though being a dimer, was found to be active, producing Hz at levels comparable to the recombinant protein (Fig. 1C), thus suggesting that in-vivo, HDP could indeed be involved in Hz production. Purification of native HDP by immunoprecipitation all but eliminated the possibility of lipids or HRP2 being present as contaminants in the native HDP fraction isolated from the parasite.

### Authentication of HDP-produced crystalline material as Hz

Parasite-produced Hz is identical to β-hematin and its unique and well characterized powder X-Ray Diffraction (XRD) pattern serves as its signature [3]. To authenticate that HDP-produced crystalline material truly represents Hz, it was subjected to XRD analysis. Diffraction pattern of HDP-produced crystallites was dominated by an intense reflection at 7.35° 2θ (Fig. 1D) and the entire profile matched the pattern calculated from the previously published profile of β-hematin [3]. This confirmed that the HDP-produced substance crystallizes in the same unit cell and structure as Hz. We also evaluated the size and morphology of HDP-produced Hz crystals by scanning electron microscopy. HDP-produced Hz was essentially constituted of randomly stacked crystallites of 0.1–0.2 µm in length and approximately 0.05 µm in width (Fig. 1D-inset). Hz crystallites of similar dimensions are found in parasite's FV [8].

### HDP is functionally conserved across *Plasmodium* genus

We identified HDP orthologs in seven other species of *Plasmodium* [20],[21] and its homologs in two species each of *Theileria* [22] and *Babesia* [21] and in *Toxoplasma gondii* [23] genome (Fig. S2). The protein showed 60% sequence identity within the *Plasmodium* genus, but had considerable divergence outside, as the overall sequence identity was less than 15%. We subsequently evaluated if the function of HDP is conserved across *Plasmodium* genus, by comparing the activities of HDP with its ortholog from *P. yoelii*, a mouse malaria parasite. Coding sequence of *P. yoelii* HDP (PyHDP) was amplified by RT-PCR, cloned, expressed in *E. coli* and the protein was purified to homogeneity (data not shown). PyHDP generated Hz at levels indistinguishable from its *P. falciparum* ortholog (Fig. 1E), suggesting that the functionality of HDP could indeed be conserved across *Plasmodium* genus.

### Intact HDP is required for Hz production

The carboxyl terminus region (amino acids 88-205) of *Plasmodium* HDP sequences have homology (e-value 3e-10) to fasciclin-1, an ancient and highly diverse adhesive domain, present in proteins of prokaryotic [24] and eukaryotic [25] origin (Fig. S2 and Fig. S1B). To check if this domain alone is responsible for the Hz producing activities we prepared two truncated versions of HDP (Fig. S1B). HDP3 encoded amino acids 88-205 of the full-length protein, representing the fasciclin domain, while HDP2 encoded amino acids 1–87 and lacked the fasciclin domain (Fig. S1B). The two constructs were expressed in *E. coli* and the purified recombinant proteins (Fig. S1C,D) were evaluated for their propensity to produce Hz (Fig. 1F). We found that both, HDP2 and HDP3 were unable to produce Hz, suggesting that fasciclin domain alone is incapable of synthesizing Hz and an intact protein is required for this activity (Fig. 1F). As part of the purification strategy, HDP and its truncated versions also encode a hexa-histidine tag (His_6_-tag). The Hz activity found in the full-length HDP could not be attributed to the presence of the polyhistidine tag as both HDP2 and HDP3 also encoded the tag but were unable to produce Hz and a recombinantly produced *P. falciparum* circumsporozoite protein containing a polyhistidine tag at its carboxyl terminus did not produce Hz. Interaction of full-length HDP with heme produced a Soret peak at 414 nm, whose intensity increased with an increase in concentration of heme in the reaction (Fig. 1G). In contrast, HDP2 and HDP3 showed 60–75% decrease in their capacity to bind heme, in comparison to the full-length protein (Fig.1G). Thus, the absence of Hz production activity by the two truncated versions was due to a substantial decrease in their potential to interact with heme. Heme-binding activity of HDP was not associated with His_6_-tag, as the tag alone showed negligible binding (to heme). Thus, a full-length HDP is required for heme binding and Hz production activities of the protein.

### HDP-mediated Hz production requires acidic condition

In vivo, Hz formation occurs in an acidic (pH 4.5–5.2) milieu [26] of the FV. Therefore, we investigated the optimal pH requirement for the activity of HDP. We found that HDP produced Hz at pH 5.2 or lower (Fig. 1H), showing complete loss of activity with an increase in pH.

### Effect of chloroquine on HDP-mediated Hz production

We subsequently tested the effect of chloroquine (CQ), a known antimalarial drug that acts by binding to heme [7],[8], on HDP-mediated Hz production. We found that the reaction was inhibited with an IC_50_ of 11 µM (Fig.1I). However, this inhibition was due to the interaction of CQ with heme, as the drug had no measurable interaction with HDP (data not shown).

### HDP is thermostable in nature

**Figure 2:**
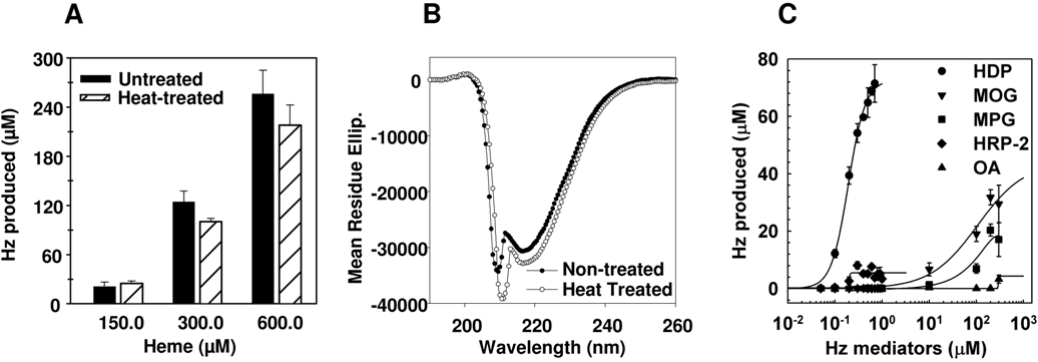
Structural and biochemical analysis of HDP-mediated Hz formation. A, HDP (Black bars) retained its Hz production activity after heat treatment (Hashed Bars) at 94°C for 10 minutes. B, CD spectra of heat-treated HDP is qualitatively similar to non-treated HDP, which was incubated at room temperature. Mean residue ellipticity is expressed as degree cm^−1^ dmol^−1^. C, When compared to MPG (square), MOG (inverted triangle), HRP 2 (diamond) and OA (triangle), HDP (circle) was most effective in producing Hz.

Parasite extracts are known to retain their Hz production activities even after boiling [13]. To evaluate if HDP could be contributing towards this phenomenon, we incubated HDP at 94°C for 10 minutes and subsequently evaluated its propensity to produce Hz. The protein produced Hz, with no measurable decrease in activity with respect to control that was incubated at room temperature for 10 minutes (Fig. 2A); thus suggesting that HDP could have been the thermostable moiety present in the extracts. Structural analysis of the heat-treated protein by circular dichroic spectroscopy showed a spectrum that was qualitatively similar to the untreated protein, thus suggesting an inherent thermostability within the HDP structure (Fig. 2B).

### HDP is the most potent Hz producer in *Plasmodium* parasites

In vitro, histidine rich proteins [10] of *P. falciparum* and neutral lipids [12],[17],[18] can produce Hz. We compared the potency of HDP with the Hz production activities of Histidine Rich Protein-2 (HRP2), Monopalmitic glycerol (MPG), Mono-oleoyl glycerol (MOG) and Oleic acid (OA). We varied the concentrations of these mediators while keeping the substrate (heme) constant at 300 µM, giving rise to molar ratios widely utilized in previous studies [10],[12]. Hz production increased, in a concentration dependent manner, with an increase in concentration of HDP, MPG and MOG in the reaction (Fig. 2C). HDP-mediated production rose rapidly, producing 71 µM of Hz at the highest concentration of protein (0.6 µM) tested. In contrast, at the highest concentration of MPG and MOG (300 µM) tested, which was equal to the concentration of heme in the reaction, 17 and 30 µM of Hz was produced, respectively. In comparison, only 0.1–0.2 µM of HDP (Fig. 2C) was required to produce the same amount of Hz. Thus, HDP was 1500–2000 folds more efficient than MPG and MOG in converting heme into Hz. At their highest concentration, HRP2 and OA could only produce a maximum of 8 and 4 µM of Hz, respectively (Fig. 2C). Negligible amounts (<0.1 µM) of Hz was produced in the absence of any of these mediators.

### Intracellular Localization of HDP reveals a circuitous trafficking mechanism

**Figure 3:**
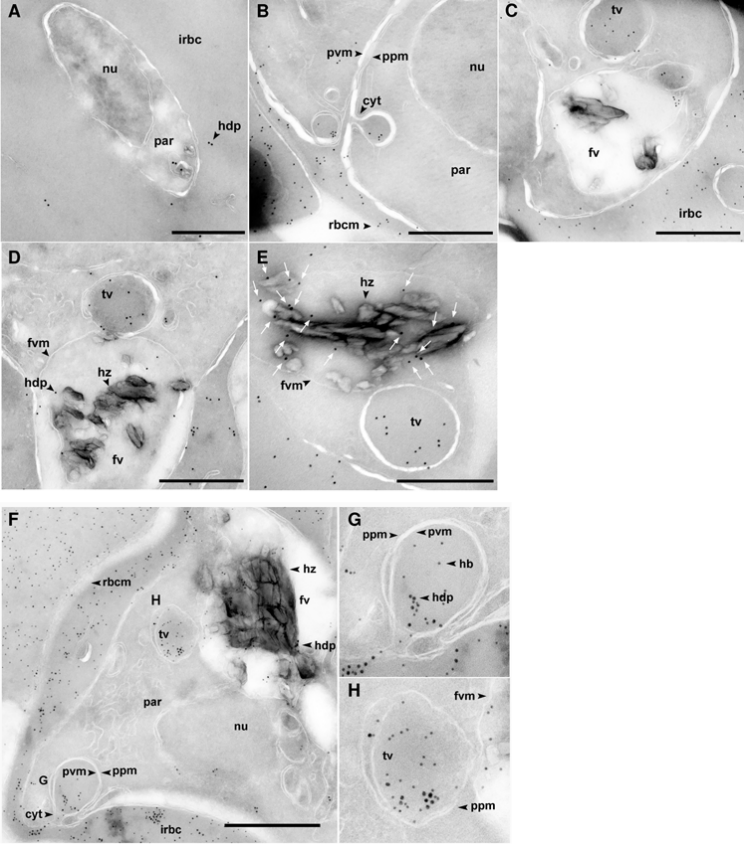
Circuitous transport and delivery of HDP to the FV. A, HDP is secreted into the cytosol of infected erythrocytes (arrowhead) in early ring stages before any Hz could be detected inside the parasite. B, After secreting it into the host cell cytosol, parasite intakes HDP through the cytostome. C, HDP could be found in transport vesicles destined to the FV. D, Transport vesicles deliver HDP to the FV. E, HDP (arrows) was found in close proximity of Hz crystals. F, HDP (18 nm particles) is transported to FV along with hemoglobin (12 nm particles). Inset G, HDP is being internalized along with hemoglobin. Inset H, Transport vesicle ready to deliver both, HDP and hemoglobin, to the FV. cyt, cytostome; fv, food vacuole; fvm, fv membrane; hz, hemozoin; hdp, heme detoxification protein; hb, hemoglobin; nu, nucleus; par, parasite; ppm, parasite plasma membrane; pvm, parasitophorous vacuole membrane; irbc, infected red blood cell; rbcm, RBC membrane; tv, transport vesicle. Bar, 0.5 µm.

As Hz formation occurs inside the FV, to be functionally relevant, HDP should be present inside this organelle. Though the protein lacks a classical N-terminal signal sequence or any known targeting signal [27],[28] that could predict its possible sorting and transport to its destined site, we detected the presence of HDP, in close proximity of Hz, within the FV (Fig. 3E), where it co-localized with Plasmepsin II, a protease present in the food vacuole (Fig. S3C–E). Therefore, to comprehend its trafficking, we analyzed its expression through intraerythrocytic stages of development in *P. falciparum* parasites. We discovered that from the early (ring) stages of infection, HDP is secreted to the host cell cytosol, before any detectable amount of Hz was visible inside the parasite (Fig. 3A). The protein accumulated inside the infected RBC cytosol (Fig. 3B,F) and was not exported out of the infected RBC, as it could not be detected in the concentrated culture supernatant by immunoblot (data not shown). Subsequently, as parasite development progressed, we found that HDP (Fig. 3B), along with host hemoglobin, is trafficked to the FV, via the cytostome-mediated pathway (Fig. 3F). We detected the uptake of HDP through the cytostome (Fig. 3B,G), its presence in the transport vesicles (Fig. 3C,H) and subsequent delivery to the FV (Fig. 3D), where large amounts of this protein could be found in close proximity of the Hz crystal (Fig. 3E and Fig. S3D,F). The recognition of HDP by the antibodies was specific, as >95% of the immuno-gold reactivity was lost when antibodies were pre-incubated with the recombinant protein (Fig. S4) and they also did not recognize un-infected RBCs (data not shown). The trafficking of HDP to the cytosol of the infected RBC did not occur through a classical secretory pathway as it could not be blocked when early ring stage parasites were treated with Brefeldin A (Fig. S5).

**Figure 4:**
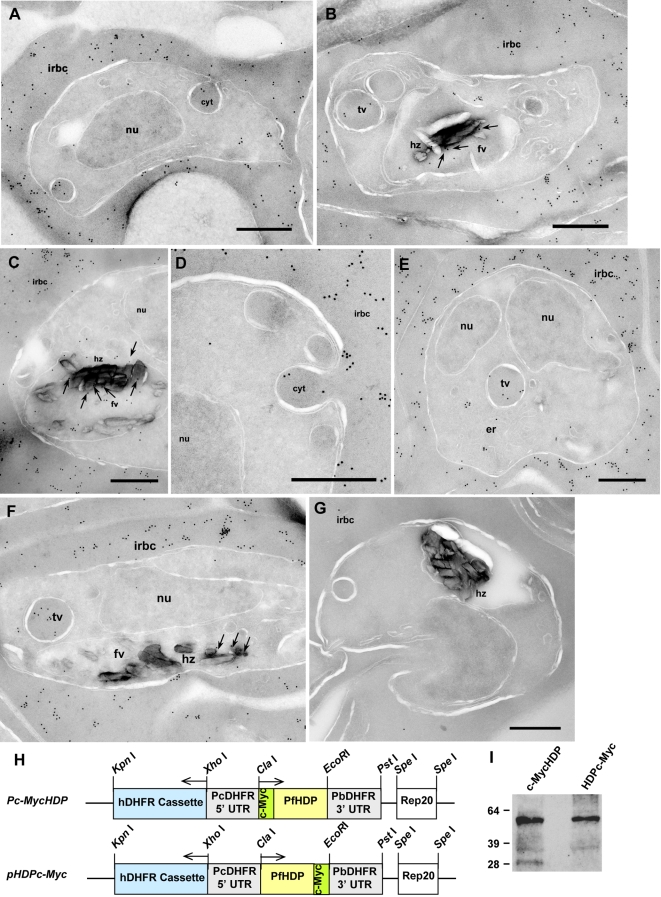
c-Myc tagged HDP has an identical trafficking pathway to HDP. Panels A–C depicts intracellular location of c-MycHDP fusion protein in parasite-infected RBC. Cytostomal uptake of c-MycHDP (A) followed by its transport (B) and its presence within the FV (C) in close proximity of Hz (shown by arrows). Panels D–F show intracellular location of HDPc-Myc, where, like HDP, chimera was present in the infected RBC cytosol (D), detected in the process of being internalized (D), transported (E), and found in the FV (F), where it presence has been marked with arrows. G, c-Myc antibodies specifically recognized the two chimeras as they did not recognize any protein in the non-transfected 3D7 *P. falciparum* parasites. H, Schematic representation of the two c-Myc based fusion constructs utilized for expression. I, c-myc tag was still attached to HDP when the protein was secreted out into the infected RBC cytosol. HDP was primarily present as a dimer, with little monomeric form also detected in c-MycHDP preparation. cyt, cytostome; fv, food vacuole; hz, hemozoin; nu, nucleus; irbc, infected red blood cell; tv, transport vesicle. Bar, 0.5 µm

This novel and circuitous trafficking route has never been observed for any of the known *Plasmodium* proteins. Hence, to validate this routing, we developed genetically modified parasite lines that episomally expressed HDP as a c-Myc tagged fusion (Fig. 4). Optimal trafficking requires recognition of specific motifs in the target protein [27] and can be affected if a tag is present in close proximity of the targeting signal [29]. As HDP does not encode any of the known targeting signals identified in *Plasmodium* [27],[28], the optimal site for the attachment of c-Myc tag could not be predicted. Therefore, we developed both, amino (c-MycHDP) and carboxyl (HDPc-Myc) termini fusions for investigating its trafficking (Fig. 4H). Upon expression, we found that both, c-MycHDP (Fig. 4A) and HDPc-Myc (Fig. 4D), were secreted into the cytosol of infected RBC. This not only validated our previous observation that HDP is exported into the infected RBC cytosol but also suggested that the protein encodes a hitherto unidentified targeting signal, which facilitates its export to the host cell cytosol. HDP does not undergo any proteolytic processing during its export as the c-Myc tag, attached to either end of the protein, was found to be intact (Fig. 4I). Like native HDP, the “inbound trafficking” of the two tagged fusions could be seen via their cytostomal uptake (Fig. 4A,D) and vesicular transport (Fig. 4B,E) that led to the delivery of fusion proteins to the FV, where they could be found in close proximity of Hz (Fig. 4C,F). The recognition of the two tagged fusions (by anti c-Myc monoclonal antibodies) was specific as wild-type 3D7 parasites showed no immunogold staining (Fig. 4G). Thus, c-Myc based fusions corroborated that HDP has an unusual trafficking route that has never been observed for any of the known *Plasmodium* proteins (schematic model depicted in Fig. S6).

### Measurement of the amount of HDP produced by the parasite

Western blot analysis of the trophozoite-infected RBC extract revealed that HDP was present predominantly as a dimer, while the FV preparation showed both the monomeric and dimeric form of the protein (Fig. S7A). The presence of HDP in the FV was not due to a contaminating cytosolic fraction, as the preparation was negative for lactate dehydrogenase, a cytosolic enzyme (Fig. S7G). Due to a continuous influx of HDP to the FV (Fig. 3E,F), its concentration in this organelle cannot be accurately measured. However, as the protein is first secreted into the RBC cytosol, by using infected RBC extracts we measured total HDP produced by the trophozoites by dot blot analysis (Fig. S7C–E) followed by a densitometric quantitation (Fig. S7F) using ImageQuant software (GE Biosciences). Intensity (in arbitrary units) of the spots representing infected RBC extracts was plotted against the intensity from the defined amounts of HDP. Albeit semi-quantitative, it allowed us to estimate the relative amount of HDP produced by the parasite. Though hemoglobin levels in an infected RBC will not increase, our measurement, being a snapshot of HDP levels that could be attained inside an infected RBC, does not take into the account any subsequent production or degradation of HDP that could have occurred during the trophozoite stage of development. The analysis revealed that 1 million parasites express approximately 40 fmol of protein (Fig. S7C–F). Therefore, each trophozoite could be producing up to 40 zeptomoles (zmol) of HDP. As an erythrocyte has a volume of 0.1 picoliter, concentration of HDP inside an infected RBC could reach 0.4 µM.

### HDP gene locus cannot be mutated

**Figure 5:**
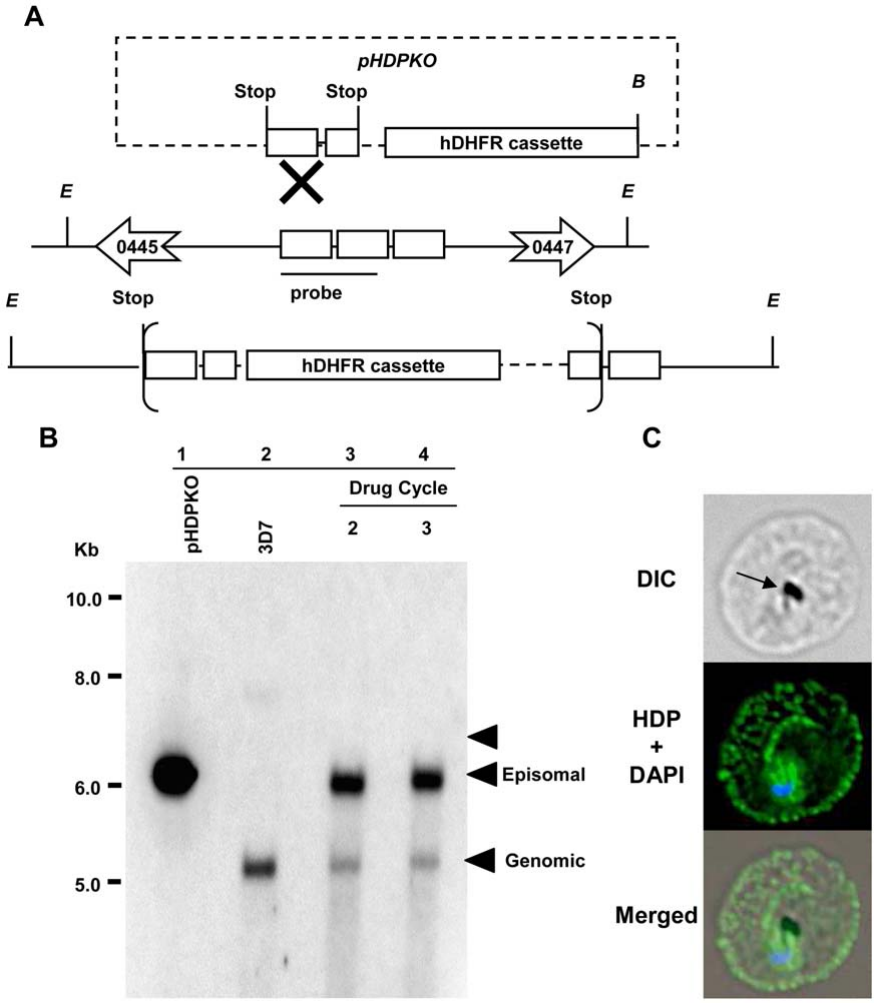
Genetic manipulation of HDP locus. Schematic representation of the strategy used for targeting *HDP* locus through single cross over recombination. A, Schematic representation of the plasmid and the genomic locus showing the enzyme sites and the genes flanking *HDP*. The anticipated cross-over event at the *HDP* locus and restriction enzyme sites for *Eco* RV, *E* and *Bam* HI, *B* are shown. *Eco* RV site is unique to the parasite DNA while *Bam* HI site is unique to the plasmid. B, Lanes 1 and 2, depict *Bam* HI-linearized *pHDPKO* (6.3 kb) and *Eco* RV and *Bam* HI digested DNA from wild type *P. falciparum* parasites containing the *HDP* locus (5.3 kb), respectively. Lanes 3 and 4 depict the transfected parasites after 2^nd^ and 3^rd^ drug cycle, respectively. Parasites surviving after three selection cycles (lanes 3, 4) had an intact *HDP* locus and an episomal copy of the *pHDPKO* plasmid expressing hDHFR. The expected size on integration is indicated by the arrow (6657 bp). C, Immuno-fluorescence staining using anti-HDP antibodies showed that the transfected parasites continually expressed HDP (green) even after the third drug cycle thus indicating that the locus was not disrupted. Arrows on the DIC image indicate hemozoin. Parasite nucleus (blue) is stained with DAPI.

**Figure 6:**
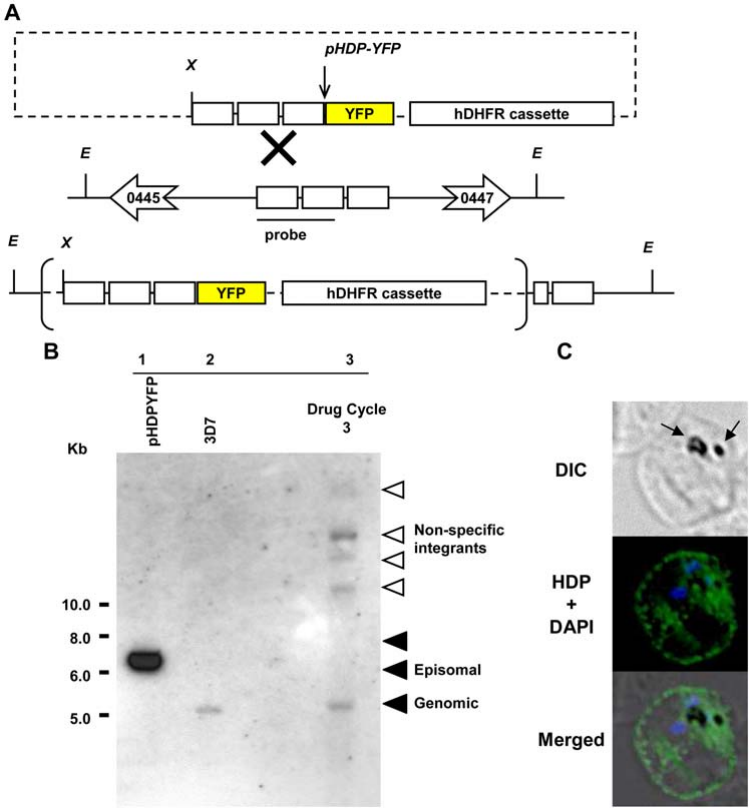
Non-specific recombination of *HDP-YFP* chimera. A, Schematic representation of the gene replacement strategy employed for obtaining HDP knocked-in parasites. The anticipated cross-over event at the *HDP* locus and the restriction enzyme sites for *Eco* RV, *E* and *Xho I*, *X* are shown. B, Lanes 1 and 2 depict *Xho* I linearized *pHDP-YFP* (6.32 kb) and *Xho* I and *Eco* RV digested DNA from wild type *P. falciparum* parasites containing the *HDP* locus (5.3kb), respectively. Lane 3 depicts the double digested DNA from transfected parasites after the 3^rd^ drug cycle. Integration at non specific sites was observed as seen by higher size bands in comparison to the episomal plasmid, represented by the open arrows. As the genomic DNA was digested with restriction enzymes unique to the flanking regions of the *HDP* locus (*Eco* RV) and the plasmid (*Xho* I) these bands do not appear to be site specific integrations of concatameric forms of *pHDP-YFP*. No integration was observed at the expected size as indicated by the arrow at 7588 bp. Parasites surviving after three selection cycles (lane 3) had the non-recombined *HDP* locus and no YFP expression, as determined by fluorescence microscopy. C, Immuno-fluorescence staining of these parasites with anti HDP antibodies also indicated the expression of the native HDP (green) after the third drug cycle. Arrows on the DIC image indicate hemozoin. Parasite nucleus (blue) is stained with DAPI.

**Figure 7:**
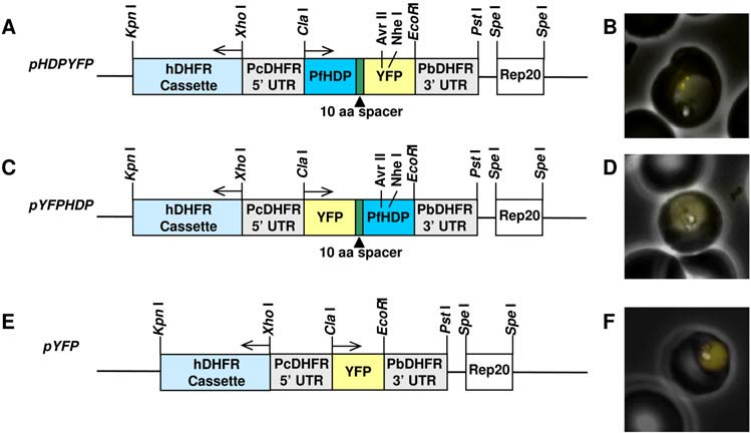
Episomal expression of the YFP-based chimera. *P. falciparum* 3D7 parasites were independently transfected with plasmids *pHDP-YFP* (A), *pYFP-HDP* (C) and *pYFP* (E) containing the hDHFR cassette as selection marker. Culture was maintained in the presence of WR92210 and the expression of yellow fluorescent protein was investigated by live cell imaging. On expression, transport of both the fusion proteins was found to be disrupted (B and D) with protein exclusively localized within the parasite cytosol. *pYFP*, when expressed alone (F) showed YFP, like GFP [50], to be localized within the parasite.

To determine if HDP is critical for parasite survival, we targeted its locus in the *P. falciparum* genome. Our two attempts to disrupt the HDP coding sequence did not produce parasite of the desired phenotype (Fig. 5A). After three selection cycles under drug pressure over a 4 month period, we could not detect site specific recombinants and the plasmid was found to be maintained episomally (Fig. 5B). Native HDP was also detectable using immunofluorescence indicating that the *HDP* locus was not disrupted (Fig. 5C). To demonstrate that this outcome is not due to an error in the transfection process, we utilized a gene replacement approach where we transfected *P. falciparum* parasites with a plasmid encoding a promoter-less full length *HDP* in fusion with the 25 kDa yellow fluorescent protein (YFP) (Fig. 6A). This would allow us to detect the emergence of transfectants and follow the trafficking of the chimeric protein [27]. While stable integrants developed after three cycles of drug selection, as confirmed by southern blot analysis, HDP locus was found to be intact, and integration of HDP-YFP cassette had occurred elsewhere in the genome (Fig. 6B). No YFP expressing parasites were visible using live cell imaging with the native HDP still detectable by immunofluorescence. This non-specific integration eliminated the possibility of any error in the transfection process and suggested that either the locus is recalcitrant to recombination or the protein chimera produced after a successful recombination event is non-functional and the resulting loss of HDP activity is detrimental for the parasite. We explored this possibility by episomally expressing HDP-YFP protein chimera in the parasite (Fig. 7A). We found that, unlike the c-Myc based fusions, the protein chimera accumulated within the parasite as the YFP-associated fluorescence was not detected in the cytosol of infected erythrocytes (Fig. 7B). This abrogation was due to YFP as its fusion at the amino terminus of HDP (YFP-HDP) also produced a similar phenotype, with fusion protein exclusively trapped within the parasite (Fig. 7C,D). YFP, when expressed alone, also remained within the parasite (Fig. 7E,F).

As both, c-Myc and YFP-based HDP fusions were expressed using identical promoter and terminator sequences, an unimpaired transport of the c-Myc tagged fusions, but, selective impairment of YFP-based fusions, indicated that the attachment of a 238 amino acids long YFP tag to a 205 amino acids HDP polypeptide has obliterated the recognition of critical motif(s) required for its export into the host cytosol. A similar phenomenon has been reported for *Plasmodium* protein RIFIN, where attachment of a GFP tag at its carboxyl terminus blocked its export into the infected RBC cytosol [29]. Thus, it is conceivable that in our gene replacement experiments, specific integration events led to the expression of HDP-YFP fusion, which could not be exported to the host cell cytosol and the resulting deprivation of the HDP-associated activities negatively influenced the survival of these genetically-modified parasites.

## Discussion

Malaria gene PF14_0446 (HDP) was one of the several hypothetical proteins selected for a malaria functional genomics study, to understand their role in malaria pathogenesis. The selection of these genes occurred through an in silico analysis, where the *Plasmodium* genome was parsed using several bioinformatical tools that predict the potential of a protein to be secreted (SignalP [30], SecretomeP [31]), possession of transmembrane domains and/or anchor sequences (TMHMM [32]), and the presence of known membrane and/or extracellular domains (SMART [33], CDD [34]). These analyses identified several hundred proteins and the feasibility aspects of cloning and expression led us to exclude sequences with low complexity regions and extremely large size (>150 kDa), which are difficult to express, purify and hence study. Specifically, for PF14_0446, it predicted that the hypothetical protein lacks a classical signal sequence and a transmembrane domain, but encodes fasciclin domain and could be secreted through the non-classical pathway of secretion. PF14_0446 (along with several other hypothetical proteins) was cloned in an *E. coli* expression vector, expressed and purified to homogeneity. The purified recombinant protein was utilized for raising antibodies, which were subsequently used for the immunolocalization studies performed on parasite-infected RBCs.

Our quest to understand the role of PF14_0446 began when we found that on expression, PF14_0446 was present in two distinct locations within the infected RBCs. This initial observation led us to further analysis, where we found that not only the protein was present in the cytosol of infected RBCs and in the FV, but it could also be detected in transit vesicles, which are responsible for the transport of host hemoglobin to the FV. Hence, we investigated the possible role of this protein in the FV and soon found that it had heme-binding properties (Fig. 1A). Though PF14_0446 has no homology to any of the known heme binding proteins, however, we found that it interacted and bound heme with high affinity (80 nM), with each polypeptide capable of binding 2.7 heme moieties. This affinity is at least 4 times higher than HRP2, whose affinity for heme is in 340–940 nM range [35]. A strong interaction of PF14_0446 with heme led us to investigate if the protein could be involved in Hz production. Using an established Hz formation assay [10], we investigated this possibility and found that the polypeptide showed robust Hz production. Hence, we named it as Heme Detoxification Protein or HDP, a label which reflects towards its putative function in the parasite.

We subsequently found that each molecule of recombinantly produced protein could convert 1566 molecules of heme into Hz hr^−1^, and the parasite-derived HDP had comparable activity. Our estimation of HDP levels in trophozoite infected RBC revealed that up to 40 zmol of HDP could be present inside the cells. As a cytosolic ingredient, 75% of the host hemoglobin is endocytosed and transported to the FV. A similar percentage of HDP, being uniformly distributed in this milieu (Fig. 3F), will presumably be transported to the FV, making 30 zmol of the protein available for Hz production. Since a mature trophozoite contains 0.55–0.6 fmol of heme as Hz [36]; with the conversion rate of 1566 molecules/hour (Fig. 1B), we believe that HDP could indeed be a playing an important role in Hz production in the parasite.

Though this rate does suggest the inherent potential of HDP in making Hz in vivo; however, the exact percentage of its contribution cannot be determined, because of an incomplete knowledge of the in vivo hemoglobin processing steps, especially the extent of proteolysis required for the release of heme (substrate) from its globin moiety, which remains unknown. Emerging evidences from several studies on parasite proteases involved in hemoglobin degradation suggests that parasite utilizes “just in time” concept to maintain its inventory of components required to undertake a systematic degradation of host hemoglobin and Hz formation [37],[38]. For example–delivery of parasite protease plasmepsin II to the food vacuole occurs along with the delivery of hemoglobin [38] and HDP, as we have shown in this study. Similarly, degradation of the inner membrane of the transit vesicle is believed to be contributing towards the pool of lipid that are subsequently found to be encapsulating the Hz. Mounting evidence from the genetic manipulation studies involving malaria proteases suggest towards redundancy, which generates an added layer of complexity for deciphering the rate at which heme could be released during hemoglobin proteolysis [39],[40]. A constant influx of the “hemoglobin processing tools” along with the substrate (hemoglobin) thus suggests that at the minimum, the rate of Hz production in the parasite is dependent on the rates at which–(i) hemoglobin and its processors are imported into the FV (ii) proteolysis of hemoglobin that leads to the release of heme (iii) influx of HDP and lipids occurs in the FV (iv) HDP and lipids act upon released heme and (v) their half life in the FV. Nonetheless, a potent in vitro Hz activity, adequate production levels, its indispensability from the parasite genome and an insight into its transport and delivery to the FV does suggest that in vivo, HDP could be an important contributor in achieving Hz levels found in the parasite.

Parasite factors responsible for Hz production have been a subject of intense debate. Slater and Cerami suggested the presence of a heme polymerase in the malaria parasite [41] and a subsequent demonstration of Hz production by HRP2 and HRP3 from *P. falciparum* parasites lead to the possibility of these proteins being the driving force behind Hz production [10]. However, (i) lack of HRP2 and HRP3 in other *Plasmodium* species, (ii) survival of *P. falciparum* parasites in their absence [42] and (iii) the secretion of most of the HRP2 into the host circulation [43], diminished their potential as the major producer of Hz in *Plasmodium* parasites. In contrast, we found HDP to be present in all the species of *Plasmodium* (sequenced to date), functionally conserved, and its locus being recalcitrant to recombination, suggesting that it could be critical for the survival of the parasite. We also found that an intact HDP was required for Hz production, indicating that the fasciclin domain encoded in the protein alone cannot produce Hz. As HDP encodes 2.7 heme binding sites and its truncated versions (HDP2 and HDP3) retained heme binding activities, albeit at much reduced levels, it suggested that heme binding involves both the regions of HDP and only an intact protein can facilitate Hz production. Interestingly, we also identified HDP homologs in *Babesia, Theileria* and *Toxoplasma* genomes; however, due to low sequence identity with members of *Plasmodium* genus, they should not be presumed as Hz producers. As HDP is also expressed at other stages of the lifecycle, it is possible that the protein could have other functions that might be in common with its homologs in *Babesia, Theileria* and *Toxoplasma* parasites.

In the parasite, a mixture of saturated and unsaturated fatty acids and neutral lipids can be found inside the food vacuole [17]. Within the food vacuole, Hz crystal is encapsulated within neutral lipids primarily composed of a mixture of mono- and diacyl glycerols [17]. In vitro, neutral lipids MPG and MOG are some of the most potent producers of Hz [12],[17] and were therefore utilized for comparison with the activity of HDP. Although micromolar conversion rates, as seen with HDP, are possible utilizing lipids, invariably, as reported by Fitch [12], Sullivan [17] and Egan [18] and shown here, these mediators are required at concentrations that are either similar [12], equal [12],[17] or in excess [18] of heme. However, only 0.15 molecules of lipid have been found to be associated with each molecule of heme [17]. We also found neutral lipids to be efficient in converting heme into Hz, albeit only when present at equimolar concentrations. In contrast, 1500–2000 fold lower concentrations of HDP could produce comparable amounts of Hz, thus suggesting that on a molar basis HDP is the most potent Hz producer in the parasite. The difference in Hz activity between lipid and HDP couldn't possibly be due to unfavorable reaction conditions for lipids as under similar conditions, Pisciotta et al found lipids to capable of rapidly producing Hz crystals [17].

The presence of 2.7 heme binding sites in the protein, a high affinity for heme and the rapid rate of conversion suggests that in vivo, HDP is involved in the formation of Hz dimer. Its involvement couldn't possibly be limited to a simple nucleation step as an increase in the concentration of either heme or HDP in the reaction leads to an increase in Hz formation. It can be hypothesized that in vivo, HDP rapidly mediates the formation of Hz dimers and possibly chaperones and delivers them to the lipid nanospheres where stacking of these dimers leads to the Hz crystal. This could potentially involve an interaction between HDP and lipids. Recently, Egan has proposed the possibility of an involvement of a protein chaperone in incorporating heme into the lipid bodies [44]. Furthermore, modeling studies also suggest that hydrogen bonding of the protonated propionic acid groups required for the final assembly of the Hz crystal is strongly favored in the hydrophobic lipid environment [18]. In evidence, >98% of the heme content in the lipid bodies was present as Hz crystals and the stoichiometry of heme:lipid found in these nanospheres [17] when utilized in vitro, produced very little Hz. Alternately, a synergistic interaction between lipids and HDP could be responsible for in vivo Hz production and remains to be investigated.

Along with a distinct biochemical activity, we found that HDP has a unique trafficking route. Though the protein does not encode any of the known host cell targeting signals [27],[28], which could facilitate its transport across the parasite plasma membrane (PM), parasitophorous vacuole (PV) or the PV membrane (PVM), we found that HDP was readily secreted into infected RBC cytosol before any Hz could be detected in the parasite; thus suggesting the presence of novel targeting signals being encoded in the parasite genome. In malaria parasite, a continuous outbound trafficking to the PM of host RBC is equally matched with a concomitant inbound trafficking of the host hemoglobin. Subsequently, in a clear demonstration of functional convergence, we found that as parasite endocytosed hemoglobin through a cytostomal-mediated pathway, HDP was also internalized and co-transported to the food vacuole. Interestingly HRP2, which is also secreted into the cytosol of infected RBC by the parasite, is not internalized by the cytostome and a direct routing of HRP2 from the parasite cytoplasm to the food vacuole has been proposed [45]. It is possible that HDP encodes signal(s) that facilitate its uptake through the cytostome. Though a mechanism, where a *Plasmodium* protein is first exported into the host cell cytosol only to be subsequently imported by the parasite machinery has never been reported for any of the known malaria proteins, the parasite does incorporates Plasmpesin II, a protease involved in hemoglobin degradation, into its plasma membrane, which during hemoglobin uptake becomes part of the cytostome and transit vesicle [38]. Thus, a cytostome-based routing seems to be the parasite's preferred mechanism for delivering contents to the food vacuole.

To our knowledge, this is the first report of a pan-*Plasmodium* heme detoxifying protein that is highly efficient in mediating the conversion of heme into Hz. As HDP had no homology to any of the known heme binding proteins, it would not have been possible to predict its role in hemozoin formation by a bioinformatic approach alone. This study is a text book example of not only the presence of non-predictable activities in a protein, but also shows that following genome sequencing of pathogens, identification of novel therapeutic targets will require experimental support by classical biochemical and cell biological approaches. Identification of HDP not only fills an important gap in our understanding of the mechanism of Hz production in malaria parasite, but the novel “Outbound-Inbound” trafficking of HDP also reveals an interesting insight into the inner workings of the parasite. Transcriptomic [19] and proteomic [46] studies of malaria parasite indicate that HDP is also expressed at mosquito and liver stages of the lifecycle, suggesting that the protein could have more than one function in the lifecycle of the parasite. Identification of new drug targets is vital for developing the next generation antimalarial drugs. With our discovery, drugs that specifically interact with HDP and obliterate its detoxification activities could potentially be developed.

## Materials and Methods

### Cloning, recombinant expression and purification of HDP

Coding sequence of HDP was amplified by RT-PCR using total RNA from the *P. falciparum* (3D7 strain) erythrocytic stage parasites. The amplified fragment was cloned in pET101, a V5 epitope and polyhistidine-tag encoding, T7 promoter-based *E. coli* expression vector, giving rise to plasmid *pHDP*. Protein, expressed in BL21 cells, was localized in inclusion bodies, which were isolated as described previously [47]. Purified inclusion bodies were solubilized in 50 mM CAPS buffer (pH 11.0) containing 1.5% N-lauryl sarkosine and 0.3 M NaCl, for 30 minutes and the solubilized protein was separated by centrifugation (10,000×g; 30 minutes). Protein was purified by affinity chromatography on His-Trap, a high performance nickel affinity column (GE Health Care) using an imidazole gradient in 50 mM CAPS pH 11.0 containing 0.3% N-lauryl sarkosine and 0.3 M NaCl. Protein-containing fractions were pooled and purified to homogeneity by gel filtration chromatography on Superdex 200 10/300 GL column (GE Health Care), equilibrated in 25 mM CAPS (pH 11.0) containing 135 mM NaCl. PyHDP was amplified by RT-PCR using total erythrocytic stage *P. yoelii* RNA and cloned in pET101 plasmid. Plasmids encoding proteins HDP2 and HDP3 were generated by sub-cloning using *pHDP* as template. Their expression and purification was performed as described above. DNA encoding *P. falciparum* HRP 2 was cloned in pET101 and its expression and purification was performed as described previously [10].

### Measurement of Binding affinity

Binding affinity of HDP for heme was evaluated by Isothermal titration calorimetry where freshly prepared heme solution was incrementally added to 5 µM HDP (in 50 mM MES, pH 5.6) present inside the ITC cell. Data was collected at 30°C at a 420 rpm stir rate using 10 µl injections of the 100 µM heme into the protein solution. The resulting measurements, delta H vs. molar ratio, were fit to a single binding site model using the MicroCal Origin analysis software.

### Hz formation assay

The assay was performed as previously described [10] with the following parameters. Unless otherwise stated all hemozoin formation assays were performed at 37°C for 1 hr in a 1ml reaction volume with 0.5 µM HDP and 300 µM heme.

#### Time Kinetics

In a 1 ml reaction, recombinant HDP at a final concentration of 0.5 µM was mixed with either 300 or 600 µM of freshly prepared heme solution. The reaction was buffered with 500 mM sodium acetate pH 5.2 and was incubated at 37°C for different time periods (1–720 minutes). The reaction was stopped by adding SDS (0.1% final concentration). Unsequestered heme was removed by repeated washing of the pellet with 2.5% SDS and 0.1 M sodium bicarbonate (pH 9.1) followed by distilled water till no soluble heme was visible in the supernatant. Hz pellet was resuspended in 1 ml of 0.1 N NaOH and absorbance was measured at 400 nm. A standard curve using different concentrations of β-hematin was prepared to quantitate the amount of heme incorporated into Hz. A reaction containing buffered heme alone was used as negative control.

#### Native vs. Recombinant HDP

Purified native and recombinant HDP (0.05 µM each) in 500 mM sodium acetate buffer pH 5.2 were incubated at 37°C with physiologically relevant concentration of freshly prepared heme for 60 minutes and the amount of Hz produced was measured as described above.

#### pH Optima

pH dependence of HDP was evaluated in 500 mM sodium acetate buffer of different pH (pH 3.2–6.0).

#### Effect of CQ

0.5 µM HDP was incubated with 300 µM heme in the absence (control) or presence of increasing concentration of CQ (5–50 µM) for 1 hour. Amount of Hz produced in reaction containing CQ was measured and compared with control reactions that did not receive any drug. Data was plotted as percentage inhibition with respect to control reactions.

#### Heat Treatment

Recombinant HDP (0.5 µM) was incubated at 94°C for 10 minutes before being evaluated in a 1hr Hz assay as described above.

#### Comparative analysis of 4 Hz producers

Increasing concentration of HDP (0.1–0.6 µM), MPG, MOG, OA (0.05–300 µM) and HRP 2 (0.05–1.0 µM) were added in a 1 ml reaction buffered with sodium acetate pH 5.2 and containing a fixed concentration (300 µM) of freshly prepared heme. The reaction was incubated at 37°C for 60 minutes followed by separation and estimation of Hz as described above. All the Hz formation assays were performed at least three times in triplicates.

### HDP-Heme interaction

HDP heme interactions were measured spectrophotometrically as described [10]. Hemin stock solution in 0.1N NaOH was added simultaneously into two cuvettes, one containing a solution of 10 µM protein (HDP, HDP2 or HDP3) or polyhistidine tag in 0.1 M sodium acetate buffer, pH 5.2, and the other containing only buffer (reference cuvette), in 2 µM increments. After each addition of heme, the samples in the two cuvettes were mixed and allowed to stand for 5 minutes. Difference in absorption spectra (over 200–800 nm range) between the reference and experimental cuvette was recorded using a spectrophotometer (GE Healthcare). Heme binding curve was constructed by plotting the change in absorbance at the Soret peak (414 nm) versus the heme concentration using the non-linear regression function in Sigma Plot Software (Systat Software Inc.).

### Immunoelectron microscopy

*P. falciparum* infected erythrocytes were purified over a magnetic column, fixed in 4% paraformaldehyde/0.1% glutaraldehyde in 100 mM PIPES/0.5 mM MgCl_2_, pH 7.2 for 1 hour at 4°C and used for immunoelectron microscopy as described [38]. Controls omitting the primary antibody were consistently negative at the concentration of gold-conjugated secondary antibodies used in these studies. Transfected parasites expressing c-Myc fusions were probed using anti-cMyc monoclonal antibodies 9E10.

### Targeted deletion of HDP

*P. falciparum* 3D7 parasites was cultured in human O+ erythrocytes as described previously. To disrupt the *HDP* genomic locus, a 509 bp locus targeting sequence beginning at the first methionine of *HDP* was PCR amplified using primers with in frame stop codons. This fragment was cloned into *pHD22Y* using the *Kpn* I restriction enzyme site to yield *pHDPKO*. The stop codons in the forward and reverse primers would prevent expression of the truncated HDP in the event of site specific recombination. Ring stage parasites at 10% parasitemia were transfected by electroporation with 100 µg of super coiled *pHDPKO* using low voltage/high capacitance conditions [48]. In an attempt to replace the genomic *HDP* locus with a *HDP-YFP* gene chimera, the plasmid *pHDP-YFP* was constructed using the *pPM2GT* plasmid as a backbone [38]. *PM2* was replaced with a targeting sequence comprising the entire promoter-less *HDP* gene using the unique *Xho* I and *Avr* II restriction enzyme sites. Subsequently, the *GFP* was replaced with *YFP* and a spacer peptide was maintained in frame between *HDP* and *YFP*, as in the original *pPM2GT* plasmid [38]. The promoter less nature of this chimera allowed detection of site specific integrants as only integrants at the *HDP* locus would express YFP. The *pHDP-YFP* plasmid was transfected into ring stage parasites as described above. All transfectants were selected in the presence of 10 nM WR99210 (a gift from Dr. Jacobus, Jacobus Pharmaceuticals, Princeton NJ) and subjected to three drug selection cycles, each consisting of 21 days of growth in absence of WR99210 followed by reselection of parasites in the presence of 10 nM WR99210. The genotypes of parasites resulting from the *pHDPKO* and the *pHDP-YFP* transfections were analyzed by probing blots of *Eco* RV-*Bam* HI and *Eco* RV-*Xho* I digested total parasite DNA, respectively with a PCR amplified 509 bp fragment of *HDP* that has been cloned in the transfection vector. The signal was generated with an Alk Phos direct labeling and detection kit as per manufacturer's instruction (GE Healthcare).

### Episomal expression of c-Myc and YFP-based chimeric protein

The vector for the transient expression of chimeric proteins was designed based on a well documented episomal segregation system reported in *P falciparum* parasites [49]. The segregation sequence, *Rep 20*, was amplified from *P falciparum* genomic DNA and cloned into the *Spe* I site of the *pBluescript SK* (+) vector to yield the plasmid *P1*. The *HDP-YFP* fusion was amplified from *pHDP-YFP* using primers with *Cla* I and *Eco* RI sites and subcloned into the above plasmid yielding *P2*. Sequence representing *Plasmodium berghei dhfr* 3′UTR was amplified from *P. berghei* strain ANKA using primers with *Eco* RI and *Pst* 1 sites and subcloned into the *P2* plasmid to yield the plasmid *P3*. The 5′UTR of *Plasmodium chaubaudi dhfr* gene was amplified from *pGFPREP2* (gift from Michael Klemba) using primers with *Xho* I and *Cla* I sites and subcloned into the plasmid *P3* to yield plasmid *P4*. The drug resistance marker *hDHFR* was amplified from the *pHHT-TK* vector and subcloned into the *P4* plasmid using the *Kpn* I and *Xho* I site to create the final vector *pHDPYFP*. Site directed mutagenesis was utilized to insert a *Nhe I* site between the *HDP* and *YFP* sequence in *pHDPYFP* such that the *HDP* sequence was flanked by *Cla I* and *Avr II* while the *YFP* sequence was flanked by the *Nhe I* and *Eco RI* restriction enzyme sites, respectively, to yield the C terminal fusion expression vector *pHDPYFP-NheI*. To generate the N terminal fusion, *pYFPHDP*, the *HDP* sequence was amplified using a forward primer with *Nhe I* site and reverse primer with *Eco RI* site. This PCR product was sub-cloned into the modified base vector *pHDPYFP-Nhe I* yielding plasmid P5. Plasmid P5 was verified by sequencing and was utilized for the second step of subcloning. *YFP* was amplified using a forward primer with the *Cla I* site and the reverse primer with the *Avr II* site. This product was then sub-cloned as a *Cla I-Avr II* fragment to yield *pYFPHDP*.

For generating the control *pYFP* expressing vector, the *YFP* sequence was PCR amplified using a forward primer with a *Cla I* site and the reverse primer with the *Eco RI* site. This product was sub-cloned into the *Cla I-Eco RI* site of *pHDPYFP-Nhe I* to yield the *pYFP* control plasmid. Primers used for all subcloning steps were designed such that all the sequences were maintained in frame thus allowing expression of the YFP alone and, the C and N terminus YFP tagged proteins.

For generating plasmids with c-myc epitope tagged at the N or C terminus of HDP, *pHDPYFP* vector was utilized as the base vector. For the N terminus fusion, the *HDP* gene was PCR amplified using a primer containing the *Cla I* site with an in frame *c-myc* sequence, and a reverse primer containing the *Eco RI* site. This product was then digested and sub-cloned into the *Cla I-Eco RI* site of *pHDPYFP* to yield the *pc-MycHDP*. Similarly for the C terminus tagged HDP, the forward primer specific to HDP containing the *Cla I* site and the reverse primer with the in-frame *c-myc* sequence with a 5′ *Eco RI* site were used for PCR amplification of *HDP* and sub-cloned into the *Cla I-Eco RI* site of the base vector to yield *pHDPc-Myc* plasmid. All the plasmids were sequenced before each subcloning step to verify the constructs. Transfections were carried out with ring stage parasites as described above and drug resistant parasites were selected using 10 nM WR99210. All the transfection experiments were performed at least two times using 2 independent clones of each plasmid. Western blots of transfected parasites were performed using monoclonal antibody against the c-myc tag.

Protocol S1 describes methods employed for X-Ray diffraction, Scanning Electron Microscopy, CD spectroscopy, Preparation of parasite extract, Purification of native HDP, Isolation of food vacuole and Estimation of HDP.

## Supporting Information

Protocol S1 Describes methods employed for X-Ray diffraction, Scanning Electron Microscopy, CD spectroscopy, Preparation of parasite extract, Purification of native HDP, Isolation of food vacuole and Estimation of HDP.
(0.09 MB DOC)

Figure S1 Cloning, expression and purification of HDP proteins. A, RT-PCR amplification of HDP coding sequence. B, Schematic representation of HDP gene structure, HDP protein and its two truncated variants, HDP 2 and HDP 3. C, Recombinantly expressed and purified HDP proteins on a 12% Coomassie stained gel under reducing conditions. D, Immunoblot of purified proteins with anti-HDP antibodies.
(3.04 MB TIF)

Figure S2 Multiple sequence alignment of HDP protein sequence. Sequences were aligned using the Clustal W algorithm. Amino acids in bold represent residues that are conserved across all the known homologs of PfHDP. Residues marked with an asterisk represent amino acid positions that are identical only in the *Plasmodium* genus. Overall, the *Plasmodium* sequences have 60% amino acid identity. Fasciclin 1 domain of PfHDP has been aligned with the consensus sequence of Fasciclin 1 domain and has an e-value of 3e-10.
(4.74 MB TIF)

Figure S3 Co-localization of HDP and Plasmepsin II antibodies. Images A–F Represent a cross section of 0.20 µm thickness obtained by deconvolution microscopy. A, Differential Interference Contrast (DIC) image of a purified food vacuole with a visible Hz crystal. Arrows indicate the food vacuole membrane. B, FV preparation was free from nuclear contaminants as no staining with DAPI was observed. C, Localization of plasmepsin II. D, HDP was present within the food vacuole. E, HDP and Plasmepsin II show co-localization (yellow regions marked with white arrows) within the FV. F, Merged image of DIC and Fluorescence showing the presence of HDP and PMII around hemozoin. Bars represent 1 µm, with the average size of the FV determined to be 2.0–2.3 µm in diameter.
(0.56 MB TIF)

Figure S4 Specificity of anti-HDP antibodies. Immunoelectron microscopy, performed as mentioned in the methods section, demonstrated the specificity of anti-HDP antibodies. A, Preincubation of anti-HDP antibodies with recombinant HDP resulted in a >95% loss of recognition of HDP in the parasite. B, Recognition of parasite-produced HDP was not affected when serum albumin was used as a non-specific ligand for anti-HDP antibodies. Scale is 500 nm.
(1.13 MB TIF)

Figure S5 Brefeldin A treatment of parasites. PfHDP transport to the host RBC cytoplasm is not perturbed by Brefeldin A treatment. The top panel is a representative of 16 hour BFA treated parasite. Similar pattern of HDP expression is consistent in all the parasites visualized and in all BFA treatment conditions. Panel A–F Represents a cross section of a parasite with A, Representing DIC; B, Nuclear staining; C, HDP staining and D, The overlaid images. Early stage control 3D7 parasites E–H Representing DIC, nuclear and HDP staining, respectively.
(4.78 MB TIF)

Figure S6 Schematic model describing the Outbound-Inbound trafficking of HDP. A, During the intraerythrocytic stages of development, HDP mRNA is translated on the ribosomes and the polypeptide is exported to the cytosol of infected RBC. Owing to the lack of any known signal sequence and host targeting signals, its trafficking within the parasite cytosol and transport across the parasite plasma membrane (PM), parasitophorous vacuole (PV) and PV membrane (PVM) occurs through a hitherto unknown mechanism. B, As hemoglobin uptake is initiated, HDP along with host hemoglobin is endocytosed by the parasite and is transported in transit vesicles to the food vacuole, where it is involved in Hz production. Nu: Nucleus; Fv: Food vacuole; iRBC, infected red blood cell.
(8.52 MB TIF)

Figure S7 Estimation of HDP levels in extracts of trophozoite stage infected RBCs (iRBC). A, Immunoblot, using anti-HDP antibodies, on protein extracts from 30.5 million iRBCs and 11.6 million FV, detected intact HDP according to its predicted size in both, dimeric and monomeric forms. Lanes 1 and 2 represent 5 and 0.5 ng of recombinant HDP, respectively; lane 3 represents total protein of trophozoite-infected RBCs; lane 4 shows protein derived from food vacuoles; and lane 5 represents iRBC cytosol. A higher molecular weight band detected in the protein extracts derived from food vacuole (lane 4) could correspond to HDP that is either associated with heme/Hz or with other macromolecules present within the food vacuole. Arrow indicates the monomeric form of protein. Absence of food vacuole associated monomeric and higher molecular weight HDP bands in trophozoite-infected RBC (lane 3) could be due to incomplete lysis of the cells during the freeze thaw process. B, Native HDP from the iRBC extract was purified by immunoprecipitation for Hz assay. Left panel-silver stained gel, right panel-Immunoblot. Molecular weight standards are represented in kDa. Absence of monomeric form of HDP in these preparations is associated with a stringent washing of immunoprecipitated protein-bead complex. Protein preparations produced under less-stringent conditions contained both the monomeric and dimeric form of the protein along with other contaminants. However, this heterogeneous preparation was also capable of producing Hz (data not shown). C, Dot blot with different concentrations of purified recombinant HDP. D, Defined number of iRBC and E, Uninfected RBCs probed with anti-HDP antibodies. F, Estimation of HDP in iRBC. Intensity (arbitrary units) of spots on dot blot of different concentrations of recombinant HDP (circle) on upper X-axis analysis, compared with the intensity of defined number of iRBC (triangle) on lower X-axis. It is estimated that 1 million parasite produce 40 fmol of HDP. G, Determination of purity of food vacuoles by Lactate Dehydrogenase assay. Extracts of food vacuoles and purified trophozoite infected RBCs corresponding to 9.7×10^6^ infected cells were assayed for lactate dehydrogenase activity. Error bars indicate standard deviation.
(6.75 MB TIF)
