# Supplementary material for: HDP—A Novel Heme Detoxification Protein from the Malaria Parasite
Source: PLoS Pathog. 2008 Apr 25;4(4):e1000053. doi: 10.1371/journal.ppat.1000053 (PMC2291572; doi:10.1371/journal.ppat.1000053)
Supplement: Protocol S1 — Describes methods employed for X-Ray diffraction, Scanning Electron Microscopy, CD spectroscopy, Preparation of parasite extract, Purification of native HDP, Isolation of food vacuole and Estimation of HDP. (0.09 MB DOC) [file ppat.1000053.s001.doc]

**Supplementary Information**

**Methods**

**Powder X-Ray Diffraction** Powder data was collected at room temperature on the Oxford Diffraction Nova diffractometer, equipped with a monochromatized Cu X-ray source giving Cu-Kα radiation (λ = 1.5405 Å and λ = 1.5443 Å). The detector was an Oxford Diffraction “Onyx” CCD camera. The data was collected as two-dimensional CCD images with a 360 degree rotation of the sample during a 5 minute exposure. The resulting images were integrated to a 1-dimensional powder pattern with the CrysAlis Pro software provided by Oxford Diffraction. Data fitting and calculation of diffraction patterns from previously reported single-crystal data was performed with the GSAS program suite driven by EXPGUI [1]. The program ConQuest was used to obtain the atomic coordinates of β-hematin from the Cambridge Structural Database.

**Scanning Electron Microscopy**  Hz was produced as described and underwent preparation for SEM as described [2]. Briefly, insoluble Hz particles obtained after removal of free heme from the reaction, by repeated washing and centrifugation processes, (as described in HDP-Hz reaction protocol) were resuspended in distilled water and deposited on polyethylenimine coated silica chips by centrifugation at 3000 x g for 10 minutes, leading to the formation of a layer on the chip surface. Chips were stained with 2% aqueous uranyl acetate solution for 60 minutes followed by a consecutive rinse in 50%, 70%, 90% ethanol for 3 minutes each. After a final rinse in 100% ethanol, sample underwent critical point drying for 90 minutes followed by a gold palladium coating. Specimen was viewed using FEI Helios Nanolab dual-beam focused ion beam/scanning electron microscopy (FIB/SEM) microscope operated at 5 kV voltage.

**Circular Dichroism spectroscopy** Spectra of a 10 µM HDP solution (Heat treated or untreated) in 20 mM acetate buffer pH 5.2, was measured in far UV range (190-260 nm) using a Jasco J-720 spectropolarimeter.

**Pre-incubation of recombinant PfHDP with polyclonal antisera**. Anti-HDP serum (100 µl) was incubated with recombinant HDP (10 µg) and separately with bovine serum albumin (10 µg) for 30 minutes at room temperature. The sera were centrifuged at 13000 rpm for 15 minutes to remove any insoluble immune complexes and used at identical dilutions in subsequent immunoelectron microscopy experiments.

**Isolation of *P. falciparum* food vacuole.** FV were isolated following protocol of Saliba et al [3]. Synchronized late trophozoite-stage parasites (4 ml packed cell volume, 8% parasitemia) were washed thrice with PBS and trophozoites were isolated by saponin (0.5 µg/ml) lysis, washed twice with 5 ml of ice-cold PBS, resuspended in 10 volumes of ice-cold water, pH 4.5, and immediately triturated four times through a 27-G 1.2 cm needle. The crude vacuole preparation was centrifuged at 13,000 rpm for 2 minutes and the pellet was resuspended in 5 ml of uptake buffer pH 7.4 (2 mM MgSO_4_, 100 mM KCl, 10 mM NaCl, 25 mM HEPES, 25 mM NaHCO_3_, and 5 mM sodium phosphate), to which 10 μl of 5 mg/ml of DNase 1 was added, and the suspension was incubated at 37° for 5 minutes. Subsequently, the suspension was spun for 2 min at 13,000 rpm and the pellet was resuspended in 100 µl of ice-cold uptake buffer followed by the addition of 1.3 ml of ice-cold 42% Percoll™ containing 0.25 M sucrose and 1.5 mM MgSO_4_, pH 7.4. The suspension was again triturated twice through a 27-G 1.2 cm needle and spun at 13,000 rpm at 4°C for 10 minutes. Purified vacuoles were isolated from a dark band at the bottom of the gradient and subjected to a second percoll gradient followed by 3 washes in uptake buffer. Purified FV were subsequently utilized for western blot analysis.

**Immunofluorescence.**  Methanol fixed smears of infected RBC at 5% parasitemia or purified food vacuole were blocked with 1% fish skin gelatin prepared in PBS (FSG-PBS) for 30 minutes and incubated with rabbit anti-HDP antibodies at 1:200 for 1 hour. After three washes of 5 minutes each with PBS, bound antibodies were detected using fluorescein isothiocyanate (FITC)-conjugated goat anti-rabbit IgG diluted to 1:200. For co-localization studies, mouse anti-HDP and rabbit anti-plasmepsin II antibodies (obtained from MR4) were used at 1:200 and 1:50 dilution respectively. Secondary labelling was performed with anti-mouse TRITC and anti-rabbit FITC antibodies, used at 1:200 dilutions. Smears were simultaneously stained with 4′, 6-diamidino-2-phenylindole (DAPI) at a final concentration of 0.1µg/ml. All primary and secondary antibody solutions were prepared in 0.1% FSG-PBS immediately before use. Slides were mounted with the anti-fade reagent (Vectorshield, KPL) and images (100× magnification) were obtained using Olympus 1X 70 inverted fluorescence microscope and a Photometrix cooled charge-coupled device camera (CH350/LCCD) driven by DELTAVISION software from Applied Precision (Seattle, WA). Images were collected in the FITC, TRITC and DAPI channels with 0.20 µm optical sections, further processed and merged using the DELTAVISION and Photoshop software.

**Brefeldin-A treatment of parasites.** MAC column purified trophozoites were cultured with fresh RBCs at 2% hematocrit and the stage progression was monitored by giemsa staining to determine the time of egress of merozoites from schizonts. BFA at 5 µg/ml was added to 3 ml cultures as the schizonts were bursting (t_0_) and 1, 2, 3 and 6 hours post bursting [4]. The cultures were incubated for 16 hours (from t_0_) to 15, 14, 13 and 10 hours respectively post bursting and slides prepared. Ethanol as vehicle control was added at a final concentration of 0.1% and slides prepared as for the BFA treated parasites. Giemsa staining was performed to determine the stage of the parasite life cycle post incubation. BFA treated parasites were arrested at the ring stage with > 90% parasites as rings. IFA was carried out as described above.

**Preparation of parasite extract.** Trophozoite stage *P. falciparum* (3D7 strain) parasites were isolated from a tightly synchronized *P. falciparum* culture using a MACS column (Miltenyi Biotec), giving rise to a highly enriched (> 95%) preparation of parasites. The parasites were counted using hemocytometer and resuspended in 100 mM Phosphate buffer, pH 7.2, containing 1X Protease Inhibitor Cocktail. The suspension was subjected to a 3 freeze-thaw cycles and was clarified by centrifugation at 15,000 x g for 15 minutes at 4^0^C. The supernatant was collected and used for the purification of native HDP and estimation of total HDP content in the parasite by immunoblot analysis. The infected RBC cytoplasm was extracted using 0.1% saponin in phosphate buffered saline, as described [5].

**Lactate Dehydrogenase assay.** Synchronized culture of *P. falciparum* 3D7 trophozoite infected red blood cells was passed through a MACS column (Miltenyi Biotec) and infected cells, at > 95% purity, were eluted from the column. The cells were counted on a hemocytometer. Different dilutions of purified infected red blood cells and 5 μl of the food vacuole extract were used to estimate the amount of hemozoin. Lactate dehydrogenase (LDH) activity was assayed in protein extracts derived from food vacuoles and a similar number of purified trophozoite infected cells. Briefly, the samples were lysed in PBS supplemented with 0.2% Triton X 100 and 1X protease inhibitor cocktail (GE health care) and LDH activity was assayed spectrophotometrically at 650 nm in a lactose oxidation assay [6].

**Purification of native HDP.** Anti-HDP antibodies were raised in rabbits and affinity purified using standard protocols. Affinity purified anti-HDP antibodies were coupled to AminoLink® Plus Coupling Gel using the Seize® Primary Immunoprecipitation kit (Pierce Biotechnology), and utilized for immunoprecipitation of native HDP from the total protein extract, as per manufacturer’s instructions. Purity of the protein was established by silver staining and by western-blot analysis using Alk-Phos substrate (KPL scientific).

**Estimation of HDP** Different concentrations of purified recombinant PfHDP and extracts representing a defined number of trophozoite-infected RBCs were vacuum blotted on a pre-wetted BA83 nitrocellulose membrane (BioRad). The membrane was blocked with 5% milk prepared in 20 mM Tris buffered saline (TBS), and subsequently incubated with affinity purified rabbit anti-PfHDP IgG antibody (1:2000) in TBS containing 0.1% Tween-20 for 1 hour. Unbound antibodies were removed by washing and the membrane was incubated further with the secondary anti-Rabbit AP conjugate (1:110000, in TBS-T, 1% milk) for 1 hour. The blot was developed using a chemiluminescent substrate (AlkPhos Substrate, KPL Scientific) as per manufacturers instructions. Quantification was performed by densitometric analysis using ImageQuant (GE Biosciences), yielding results in arbitrary units.

**Sequence of primers used for the generation of HDP-based constructs:**

Rep 20 FP: GATACCTAAACTAGTACCTAATTTAACTAAC

Rep 20 RP: AGACTTAAACTAGTAATAGTAGGACCAG

*Plasmodium* *berghei* *dhfr* 3’UTR FP: TAAAATGAATTCGGATATGGCAGCTTAATGTTCG

*Plasmodium* *berghei* *dhfr* 3’UTR RP: AGGATGACTGCAGCTACCCTGAAGAAGAAAAGTCC

*pHDPYFP*  FP: TTGTGTTATCGATATGAAAAATAGATTTTATTATA

*pHDPYFP*  RP: ATACCTGAATTCTTAACTTCCTCCTAATCC

*Plasmodium chaubaudi* *dhfr* 5’ UTR FP: TATTGTTTCTCGAGTTAGCTAATTCGCTTG

*Plasmodium chaubaudi* *dhfr* 5’ UTR RP: TTATTATTATCGATTTTGTAAGTTTTAGGTGTGT

*hDHFR* FP: GTGGGACTCGAGTATAAGGAAAT

*hDHFR* RP : TGCTAGAGGTACCTTTAATAAATATGT

YFP NheI FP: TTATATTTTGCTAGCATCAGTAAAGGAGAAG

YFP EcoRI RP: TAAACTTCCGAATTCTTATTTGTATAGTTCATC

HDP ClaI FP: ACCTAAAACTTACAAAATCGATATGAAAAATAGA

HDP AvrII RP: TAGATATGACCTAGGAAAAATGATGGGCTT

YFP ClaI FP: TTATATTTTATCGATATGAGTAAAGGAGAAGAA

YFP AvrII RP: TAAACTTCCCCTAGGTTTGTATAGTTCATC

HDP NheI FP: ACCTAAAACTTACAAAATCGATATGAAAAATAGA

HDP EcoRI RP: TAGATATGAGAATTCTTAAAAAATGATGGGCTT

c-MYC-HDPRep20 Vector Cla I FP: TCTAGAAATAATATCGATATGGAACAAAAACTCATTTCTGAAGAAGACTTGATTAAAAATAGA

c-MYC-HDPRep20 Vector Eco RI RP: GAGAGGGTTAGGGAATTCTTACAAGTCTTCTTCAGAAATGAGTTTTTGTTCAAAAATGATGGG

1. Toby B (2001) EXPGUI, a graphical user interface for GSAS. J Appl Cryst 34: 210-213.

2. Noland GS, Briones N, Sullivan DJ, Jr. (2003) The shape and size of hemozoin crystals distinguishes diverse Plasmodium species. Mol Biochem Parasitol 130: 91-99.

3. Saliba KJ, Folb PI, Smith PJ (1998) Role for the plasmodium falciparum digestive vacuole in chloroquine resistance. Biochem Pharmacol 56: 313-320.

4. Khattab A, Klinkert MQ (2006) Maurer's clefts-restricted localization, orientation and export of a Plasmodium falciparum RIFIN. Traffic 7: 1654-1665.

5. Dalal S, Klemba M (2007) Roles for two aminopeptidases in vacuolar hemoglobin catabolism in plasmodium falciparum. J Biol Chem.

6. Makler MT, Hinrichs DJ (1993) Measurement of the lactate dehydrogenase activity of Plasmodium falciparum as an assessment of parasitemia. Am J Trop Med Hyg 48: 205-210.

**Figure Legends**

**Supplementary Figure S1: Cloning, expression and purification of HDP proteins.**

**A,** RT-PCR amplification of HDP coding sequence. **B,** Schematic representation of HDP gene structure, HDP protein and its two truncated variants, HDP 2 and HDP 3. **C,**  Recombinantly expressed and purified HDP proteins on a 12% Coomassie stained gel under reducing conditions. **D,** Immunoblot of purified proteins with anti-HDP antibodies.

**Supplementary Figure S2: Multiple sequence alignment of HDP protein sequence.** Sequences were aligned using the Clustal W algorithm. Amino acids in bold represent residues that are conserved across all the known homologs of PfHDP. Residues marked with an asterisk represent amino acid positions that are identical only in the *Plasmodium* genus. Overall, the *Plasmodium* sequences have 60% amino acid identity. Fasciclin 1 domain of PfHDP has been aligned with the consensus sequence of Fasciclin 1 domain and has an e-value of 3e-10.

**Supplementary Figure S3: Co-localization of HDP and Plasmepsin II antibodies.**

Images **A-F** Represent a cross section of 0.20 µm thickness obtained by deconvolution microscopy. **A,** Differential Interference Contrast (DIC) image of a purified food vacuole with a visible Hz crystal. Arrows indicate the food vacuole membrane. **B,** FV preparation was free from nuclear contaminants as no staining with DAPI was observed. **C,** Localization of plasmepsin II. **D**, HDP was present within the food vacuole. **E,** HDP and Plasmepsin II show co-localization (yellow regions marked with white arrows) within the FV. **F**, Merged image of DIC and Fluorescence showing the presence of HDP and PMII around hemozoin. Bars represent 1µm, with the average size of the FV determined to be 2.0-2.3 µm in diameter.

**Supplementary Figure S4: Specificity of anti-HDP antibodies.**

Immunoelectron microscopy, performed as mentioned in the methods section, demonstrated the specificity of anti-HDP antibodies. **A**, Preincubation of anti-HDP antibodies with recombinant HDP resulted in a > 95% loss of recognition of HDP in the parasite. **B,** Recognition of parasite-produced HDP was not affected when serum albumin was used as a non-specific ligand for anti-HDP antibodies. Scale is 500 nm.

**Supplementary Figure S5: Brefeldin A treatment of parasites.**

PfHDP transport to the host RBC cytoplasm is not perturbed by Brefeldin A treatment. The top panel is a representative of 16 hour BFA treated parasite. Similar pattern of HDP expression is consistent in all the parasites visualized and in all BFA treatment conditions. Panel **A-F** Represents a cross section of a parasite with **A,** Representing DIC; **B,** Nuclear staining; **C**, HDP staining and **D,** The overlaid images. Early stage control 3D7 parasites **E-H** Representing DIC, nuclear and HDP staining, respectively.

**Supplementary Figure S6: Schematic model describing the Outbound-Inbound trafficking of HDP.**

**A,** During the intraerythrocytic stages of development, HDP mRNA is translated on the ribosomes and the polypeptide is exported to the cytosol of infected RBC. Owing to the lack of any known signal sequence and host targeting signals, its trafficking within the parasite cytosol and transport across the parasite plasma membrane (PM), parasitophorous vacuole (PV) and PV membrane (PVM) occurs through a hitherto unknown mechanism. **B,** As hemoglobin uptake is initiated, HDP along with host hemoglobin is endocytosed by the parasite and is transported in transit vesicles to the food vacuole, where it is involved in Hz production. Nu: Nucleus; Fv: Food vacuole; iRBC, infected red blood cell.

**Supplementary Figure S7: Estimation of HDP levels in extracts of trophozoite stage infected RBCs (iRBC).**

**A,** Immunoblot, using anti-HDP antibodies, on protein extracts from 30.5 million iRBCs and 11.6 million FV, detected intact HDP according to its predicted size in both, dimeric and monomeric forms. Lanes 1 and 2 represent 5 and 0.5 ng of recombinant HDP, respectively; lane 3 represents total protein of trophozoite-infected RBCs; lane 4 shows protein derived from food vacuoles; and lane 5 represents iRBC cytosol. A higher molecular weight band detected in the protein extracts derived from food vacuole (lane 4) could correspond to HDP that is either associated with heme/ Hz or with other macromolecules present within the food vacuole. Arrow indicates the monomeric form of protein. Absence of food vacuole associated monomeric and higher molecular weight HDP bands in trophozoite-infected RBC (lane 3) could be due to incomplete lysis of the cells during the freeze thaw process. **B,** Native HDP from the iRBC extract was purified by immunoprecipitation for Hz assay. Left panel – silver stained gel, right panel – Immunoblot. Molecular weight standards are represented in kDa. Absence of monomeric form of HDP in these preparations is associated with a stringent washing of immunoprecipitated protein-bead complex. Protein preparations produced under less-stringent conditions contained both the monomeric and dimeric form of the protein along with other contaminants. However, this heterogeneous preparation was also capable of producing Hz (data not shown). **C,** Dot blot with different concentrations of purified recombinant HDP. **D,** Defined number of iRBC and **E,** Uninfected RBCs probed with anti-HDP antibodies. **F,** Estimation of HDP in iRBC. Intensity (arbitrary units) of spots on dot blot of different concentrations of Recombinant HDP (circle) on upper X-axis analysis, compared with the intensity of defined number of iRBC (triangle) on lower X-axis. It is estimated that 1 million parasite produce 40 fmol of HDP. **G,** Determination of purity of food vacuoles by Lactate Dehydrogenase assay. Extracts of food vacuoles and purified trophozoite infected RBCs corresponding to 9.7 x10^6^ infected cells were assayed for lactate dehydrogenase activity. Error bars indicate standard deviation.
