# Supplementary figures and images for: HDP—A Novel Heme Detoxification Protein from the Malaria Parasite
Source: PLoS Pathog. 2008 Apr 25;4(4):e1000053. doi: 10.1371/journal.ppat.1000053 (PMC2291572; doi:10.1371/journal.ppat.1000053)

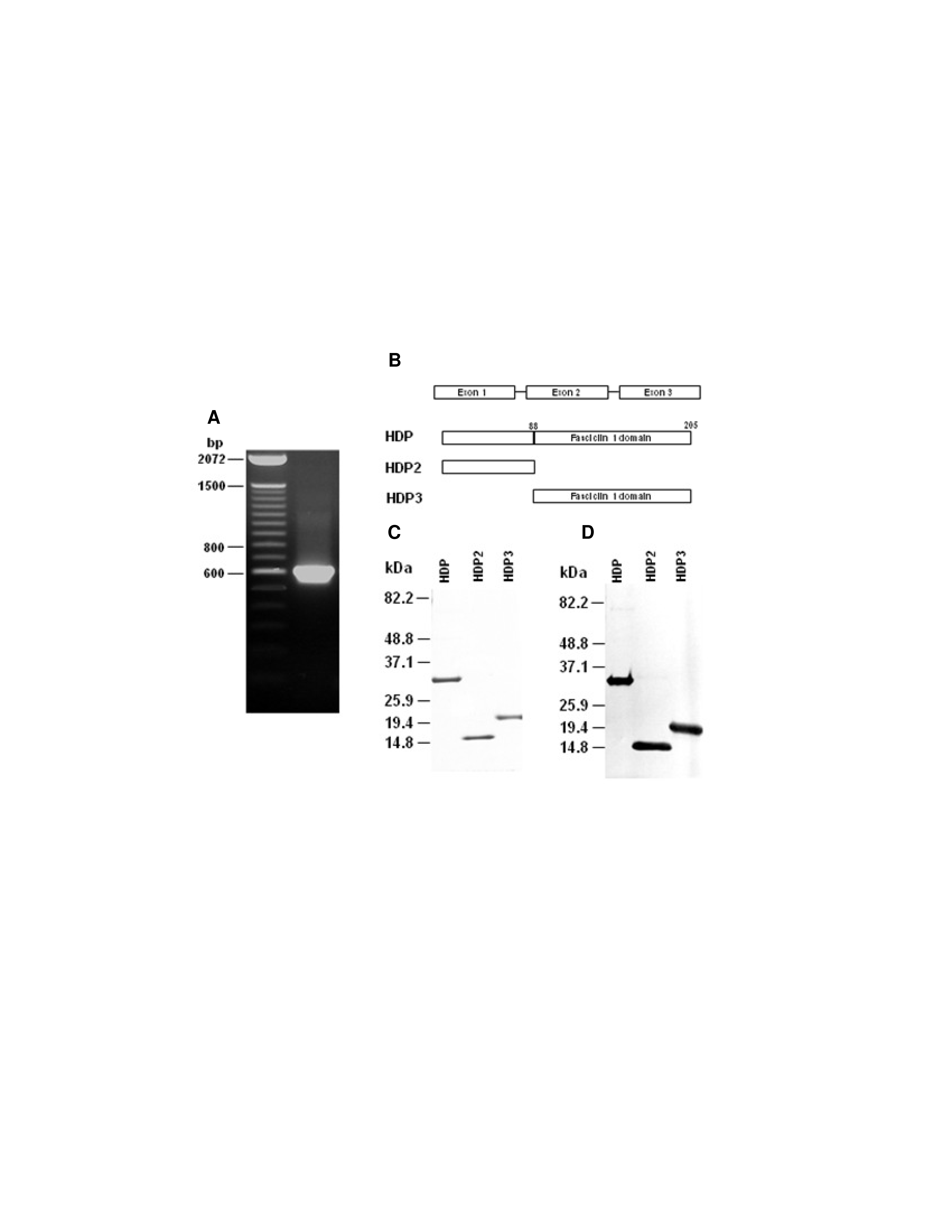

Supplement: Figure S1 — Cloning, expression and purification of HDP proteins. A, RT-PCR amplification of HDP coding sequence. B, Schematic representation of HDP gene structure, HDP protein and its two truncated variants, HDP 2 and HDP 3. C, Recombinantly expressed and purified HDP proteins on a 12% Coomassie stained gel under reducing conditions. D, Immunoblot of purified proteins with anti-HDP antibodies. (3.04 MB TIF) [file ppat.1000053.s002.tif]

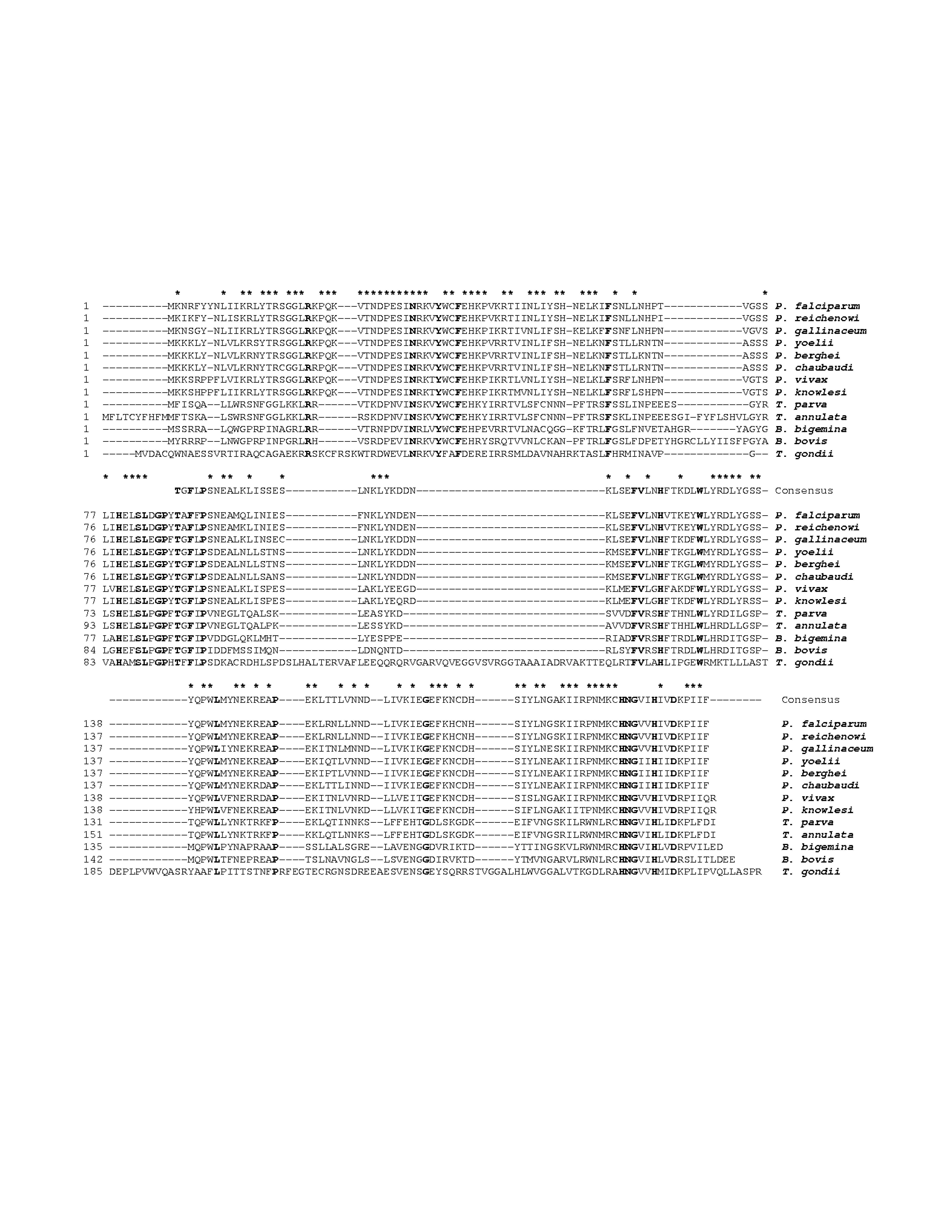

Supplement: Figure S2 — Multiple sequence alignment of HDP protein sequence. Sequences were aligned using the Clustal W algorithm. Amino acids in bold represent residues that are conserved across all the known homologs of PfHDP. Residues marked with an asterisk represent amino acid positions that are identical only in the Plasmodium genus. Overall, the Plasmodium sequences have 60% amino acid identity. Fasciclin 1 domain of PfHDP has been aligned with the consensus sequence of Fasciclin 1 domain and has an e-value of 3e-10. (4.74 MB TIF) [file ppat.1000053.s003.tif]

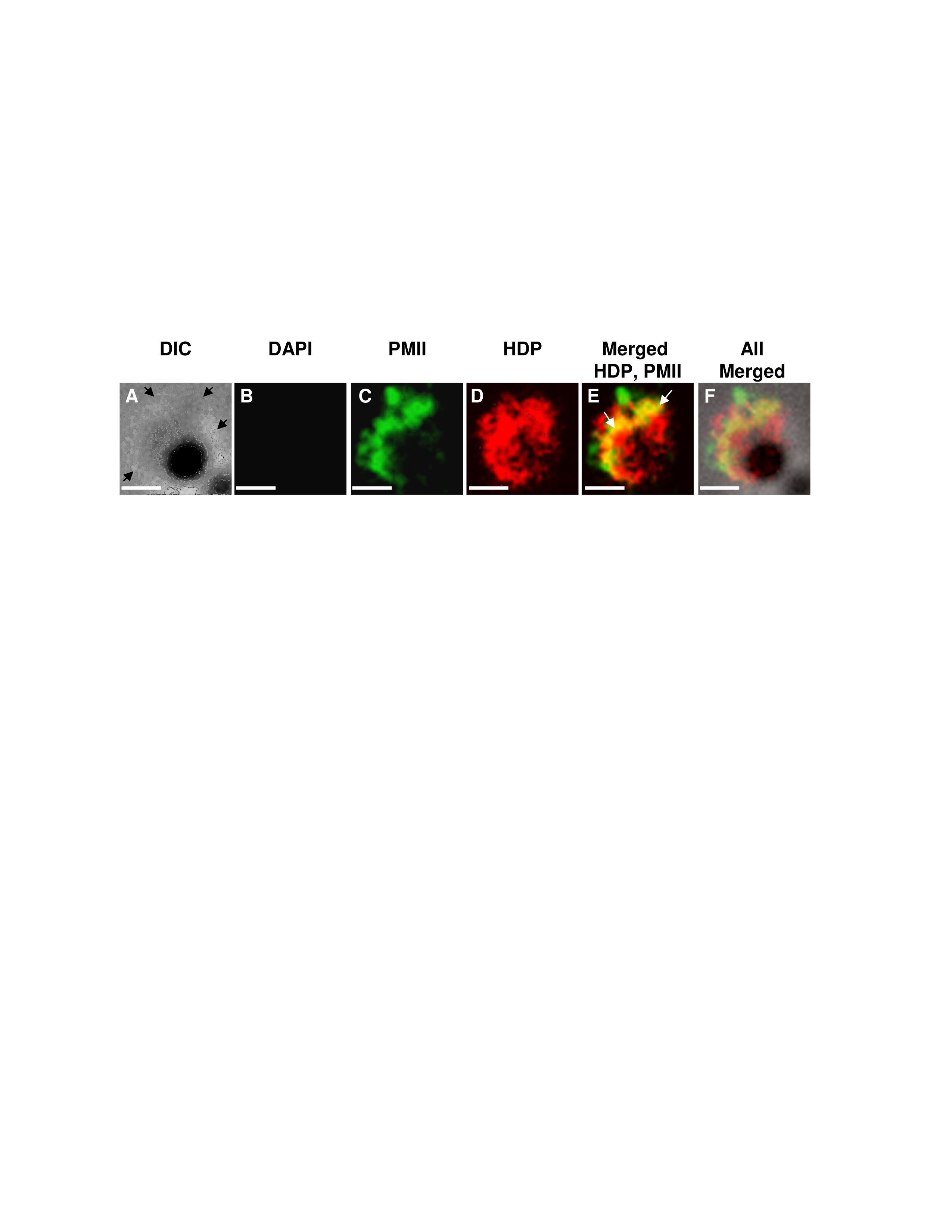

Supplement: Figure S3 — Co-localization of HDP and Plasmepsin II antibodies. Images A–F Represent a cross section of 0.20 µm thickness obtained by deconvolution microscopy. A, Differential Interference Contrast (DIC) image of a purified food vacuole with a visible Hz crystal. Arrows indicate the food vacuole membrane. B, FV preparation was free from nuclear contaminants as no staining with DAPI was observed. C, Localization of plasmepsin II. D, HDP was present within the food vacuole. E, HDP and Plasmepsin II show co-localization (yellow regions marked with white arrows) within the FV. F, Merged image of DIC and Fluorescence showing the presence of HDP and PMII around hemozoin. Bars represent 1 µm, with the average size of the FV determined to be 2.0–2.3 µm in diameter. (0.56 MB TIF) [file ppat.1000053.s004.tif]

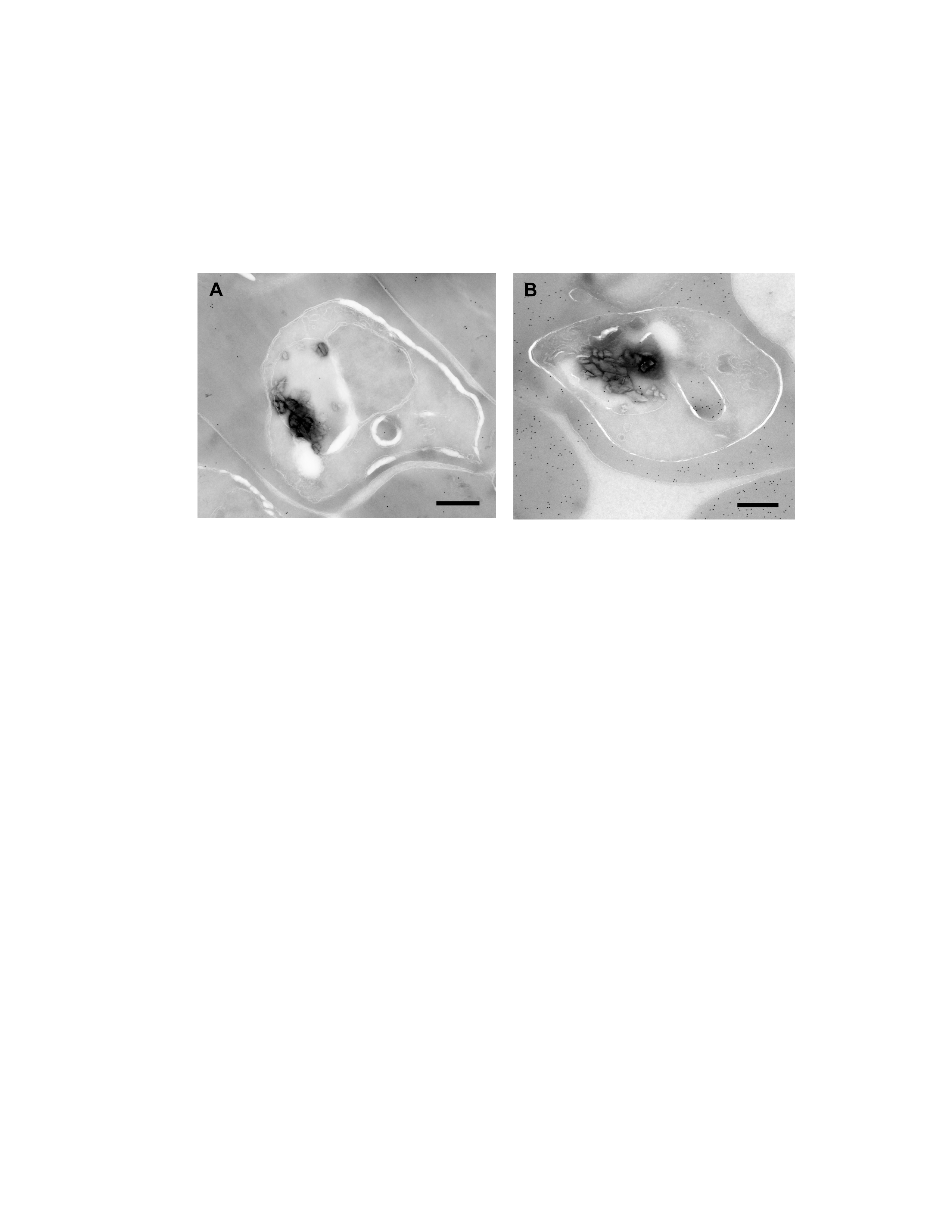

Supplement: Figure S4 — Specificity of anti-HDP antibodies. Immunoelectron microscopy, performed as mentioned in the methods section, demonstrated the specificity of anti-HDP antibodies. A, Preincubation of anti-HDP antibodies with recombinant HDP resulted in a >95% loss of recognition of HDP in the parasite. B, Recognition of parasite-produced HDP was not affected when serum albumin was used as a non-specific ligand for anti-HDP antibodies. Scale is 500 nm. (1.13 MB TIF) [file ppat.1000053.s005.tif]

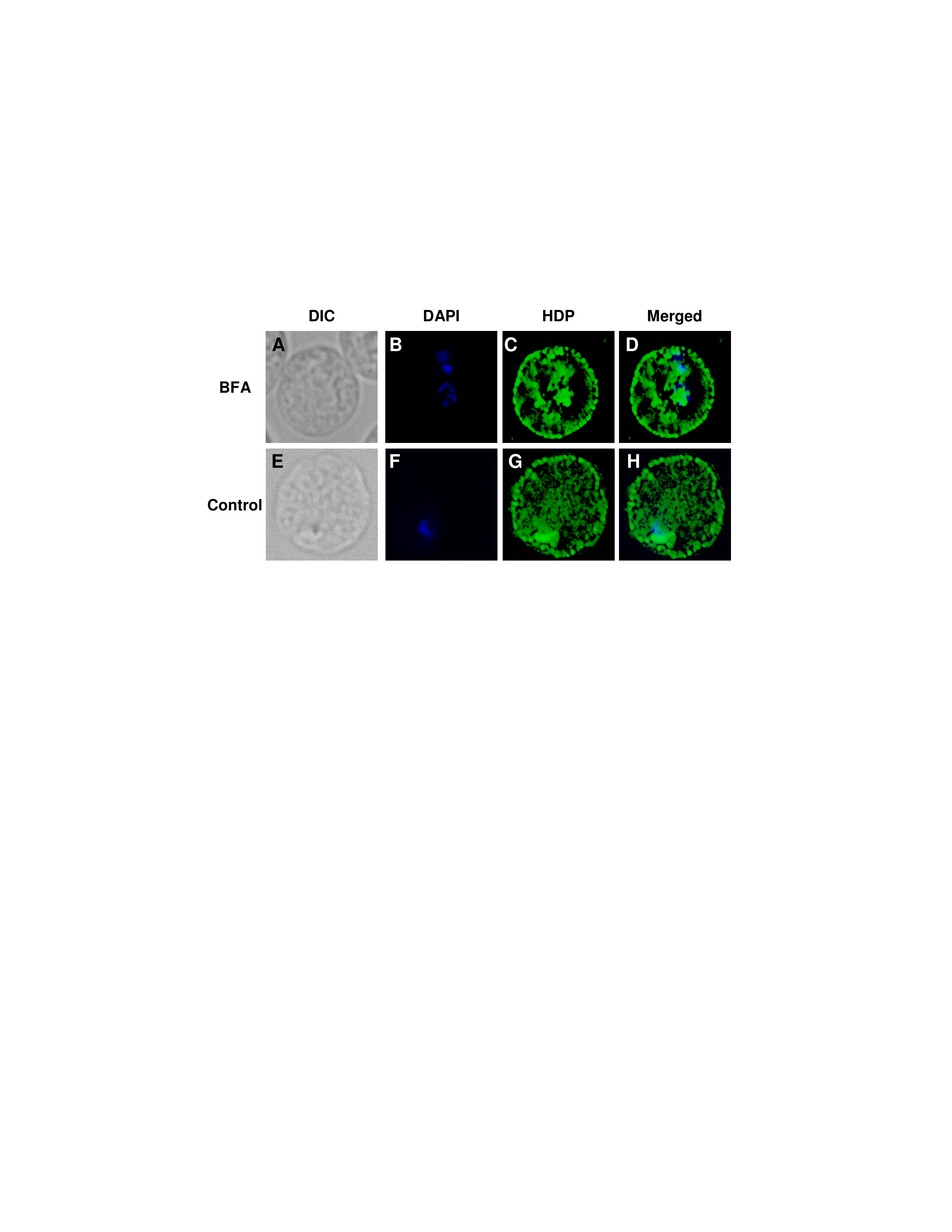

Supplement: Figure S5 — Brefeldin A treatment of parasites. PfHDP transport to the host RBC cytoplasm is not perturbed by Brefeldin A treatment. The top panel is a representative of 16 hour BFA treated parasite. Similar pattern of HDP expression is consistent in all the parasites visualized and in all BFA treatment conditions. Panel A–F Represents a cross section of a parasite with A, Representing DIC; B, Nuclear staining; C, HDP staining and D, The overlaid images. Early stage control 3D7 parasites E–H Representing DIC, nuclear and HDP staining, respectively. (4.78 MB TIF) [file ppat.1000053.s006.tif]

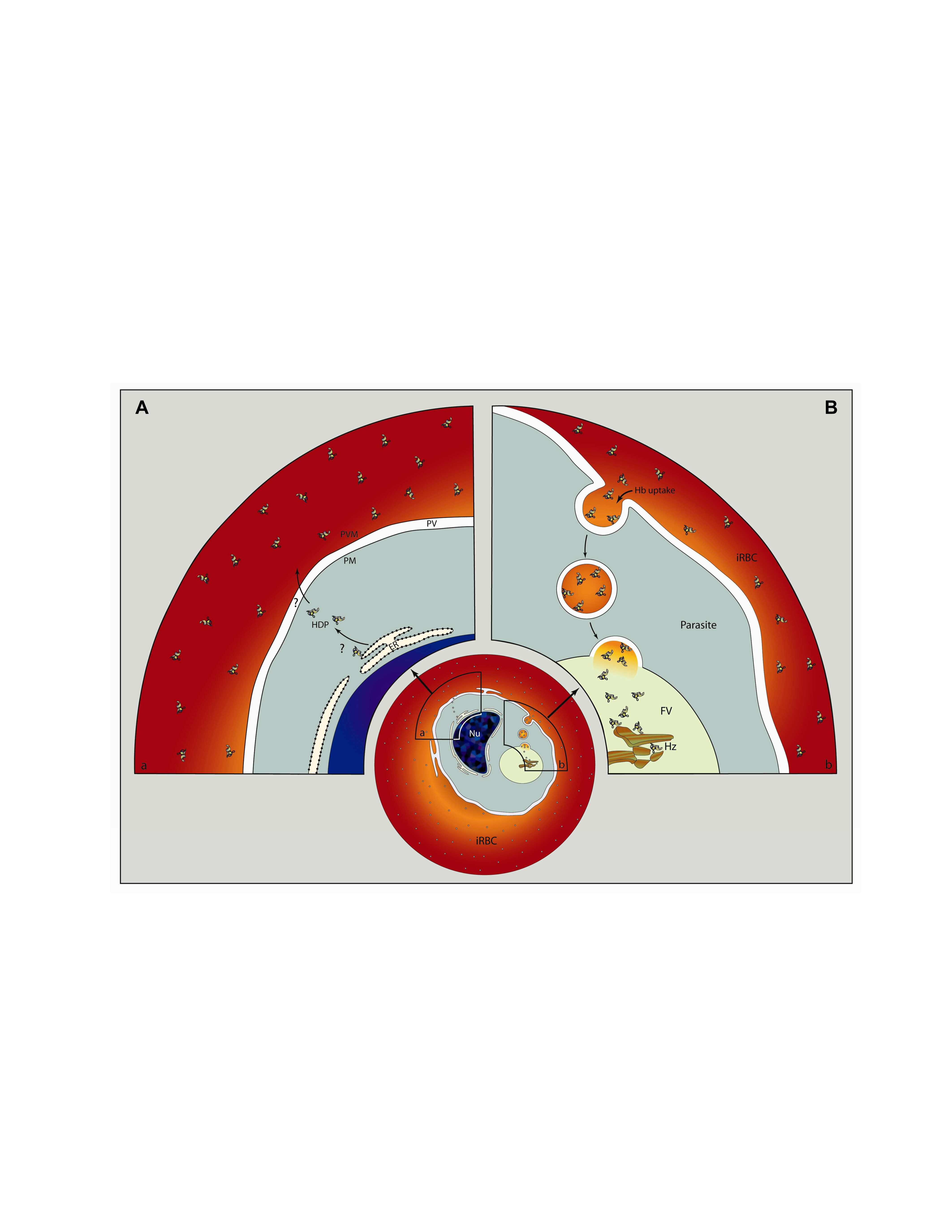

Supplement: Figure S6 — Schematic model describing the Outbound-Inbound trafficking of HDP. A, During the intraerythrocytic stages of development, HDP mRNA is translated on the ribosomes and the polypeptide is exported to the cytosol of infected RBC. Owing to the lack of any known signal sequence and host targeting signals, its trafficking within the parasite cytosol and transport across the parasite plasma membrane (PM), parasitophorous vacuole (PV) and PV membrane (PVM) occurs through a hitherto unknown mechanism. B, As hemoglobin uptake is initiated, HDP along with host hemoglobin is endocytosed by the parasite and is transported in transit vesicles to the food vacuole, where it is involved in Hz production. Nu: Nucleus; Fv: Food vacuole; iRBC, infected red blood cell. (8.52 MB TIF) [file ppat.1000053.s007.tif]

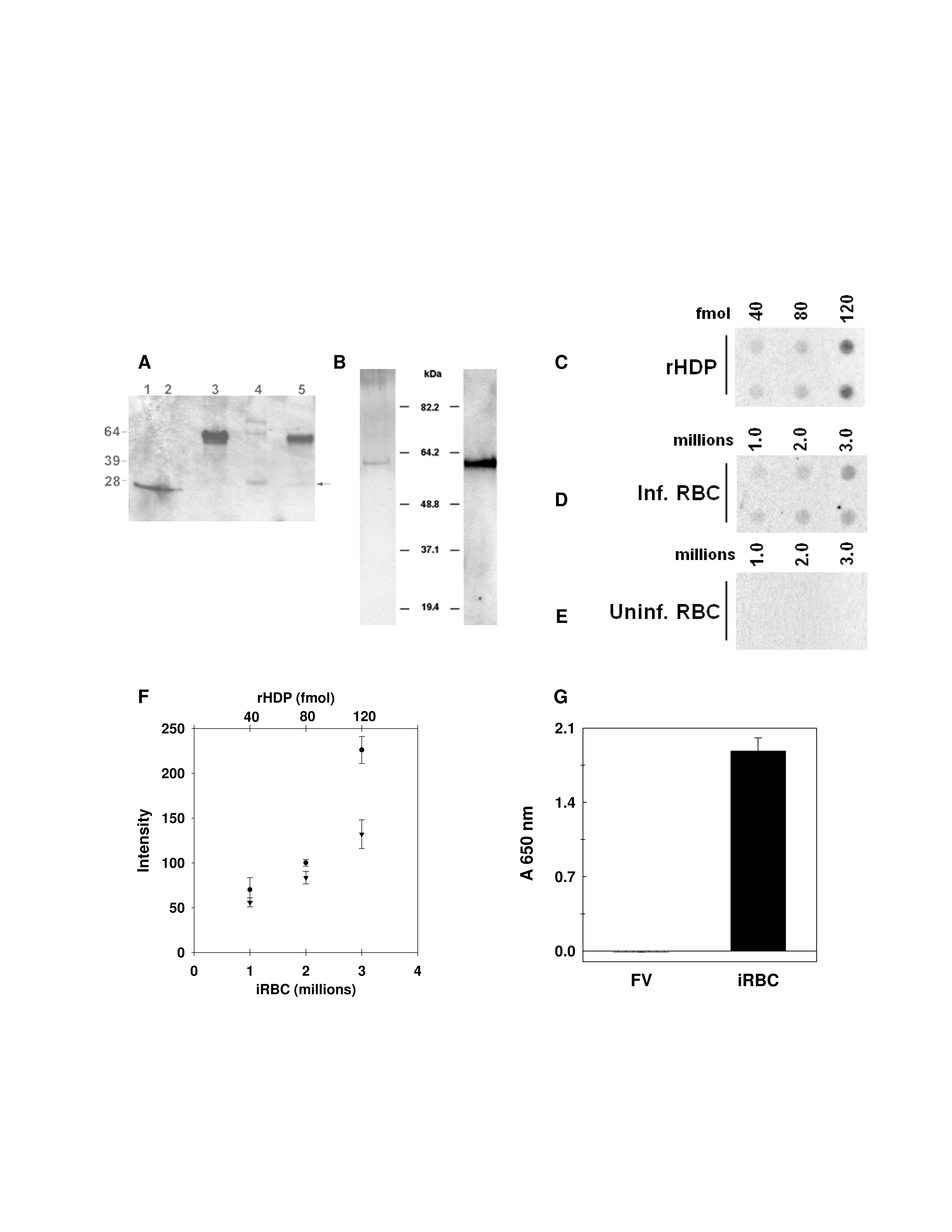

Supplement: Figure S7 — Estimation of HDP levels in extracts of trophozoite stage infected RBCs (iRBC). A, Immunoblot, using anti-HDP antibodies, on protein extracts from 30.5 million iRBCs and 11.6 million FV, detected intact HDP according to its predicted size in both, dimeric and monomeric forms. Lanes 1 and 2 represent 5 and 0.5 ng of recombinant HDP, respectively; lane 3 represents total protein of trophozoite-infected RBCs; lane 4 shows protein derived from food vacuoles; and lane 5 represents iRBC cytosol. A higher molecular weight band detected in the protein extracts derived from food vacuole (lane 4) could correspond to HDP that is either associated with heme/Hz or with other macromolecules present within the food vacuole. Arrow indicates the monomeric form of protein. Absence of food vacuole associated monomeric and higher molecular weight HDP bands in trophozoite-infected RBC (lane 3) could be due to incomplete lysis of the cells during the freeze thaw process. B, Native HDP from the iRBC extract was purified by immunoprecipitation for Hz assay. Left panel-silver stained gel, right panel-Immunoblot. Molecular weight standards are represented in kDa. Absence of monomeric form of HDP in these preparations is associated with a stringent washing of immunoprecipitated protein-bead complex. Protein preparations produced under less-stringent conditions contained both the monomeric and dimeric form of the protein along with other contaminants. However, this heterogeneous preparation was also capable of producing Hz (data not shown). C, Dot blot with different concentrations of purified recombinant HDP. D, Defined number of iRBC and E, Uninfected RBCs probed with anti-HDP antibodies. F, Estimation of HDP in iRBC. Intensity (arbitrary units) of spots on dot blot of different concentrations of recombinant HDP (circle) on upper X-axis analysis, compared with the intensity of defined number of iRBC (triangle) on lower X-axis. It is estimated that 1 million parasite produce 40 fmol of HDP. G [file ppat.1000053.s008.tif]
